# Evaluating the impact of vaping facts films on vaping harm perceptions among young adults in the UK: A randomized on‐line experiment

**DOI:** 10.1111/add.70119

**Published:** 2025-07-09

**Authors:** Mohammad Alharbi, Emma Ward, Caitlin Notley, Martin Dockrell, Eve Taylor, Katherine East

**Affiliations:** ^1^ National Addiction Centre, Institute of Psychiatry, Psychology & Neuroscience King’s College London London UK; ^2^ Lifespan Health Research Centre, Addiction Research Group, Norwich Medical School University of East Anglia Norwich UK; ^3^ Unaffiliated London UK; ^4^ Department of Behavioural Science and Health University College London London UK; ^5^ Department of Primary Care and Public Health, Brighton and Sussex Medical School University of Brighton and University of Sussex Brighton UK

**Keywords:** experiment, perceptions, smoking, social media, vaping, young adults

## Abstract

**Aim:**

Measure the impact of brief, academic‐led, evidence‐based social media videos on vaping harm perceptions among young adults.

**Design:**

On‐line between‐subjects experimental study. Participants were randomized to one of two conditions: experimental (exposed to one of eight brief videos, designed for social media, with academic experts addressing vaping harms) or control. Before and after exposure to the videos, all participants answered questions about their perceptions of vaping and smoking and socio‐demographics.

**Setting:**

Qualtrics on‐line survey platform.

**Participants:**

593 young adults aged 18–30 years who resided in the UK (49.7% female, 49.2% male; 8.9% exclusively smoked, 32% exclusively vaped, 28.7% did both and 30.4% did neither). Participants were randomly assigned to intervention (*n* = 279) or control (*n* = 314) groups.

**Measurements:**

The primary outcome was the perception that vaping is less harmful than smoking. Secondary outcomes were perceptions that vaping is harmful, vaping is addictive and responses (true, false) to statements that were matched to the videos (e.g. vaping causes cancer, vaping causes lung injuries).

**Findings:**

After exposure to an expert video, compared with those in the control group, participants in the intervention group had over three times the odds of perceiving vaping as less harmful than smoking [82.1% vs 57.6%; adjusted odds ratio (AOR) = 3.69; 95% confidence interval (95% CI) = 2.49–5.47; *P* < 0.001]. Perceptions that the following statements are false were also higher after viewing expert videos than control videos: vaping causes lung injury, vaping leads to cancer, nicotine is harmful when used in ways that does not involve smoking tobacco, pregnant women should not vape, vaping will not help you quit smoking, vaping has no place on the NHS (all *P* < 0.05). Participants exposed to the ‘vaping is as harmful as smoking’ misconception video had the highest odds of accurately perceiving vaping as less harmful than smoking (AOR = 13.92; 95% CI = 3.26–59.37; *P* < 0.001). Videos specifically targeting individual misconceptions (e.g. ‘vaping causes lung injury’ or ‘vaping causes cancer’) were particularly effective in improving related perceptions, indicating that the videos functioned as designed. There was little evidence of associations between condition and perceiving that vaping is not harmful (AOR = 2.57; 95% CI = 0.78–8.52; *P* = 0.122) or not addictive (AOR = 0.49; 95% CI = 0.04–6.67; *P* = 0.594). Findings were similar among young adults regardless of vaping and smoking status.

**Conclusion:**

Brief, academic‐led, vaping facts films appear to be effective in correcting vaping misperceptions and dispelling common misconceptions.

## INTRODUCTION

Evidence is clear that vaping is less harmful than smoking but is not without risks [1, 2, 3]. E‐cigarettes are also an effective smoking cessation tool and have been found to be more effective than nicotine replacement therapies such as patches and gums [4]. However, many people inaccurately perceive vaping e‐cigarettes to be equally or more harmful than smoking cigarettes, and these misperceptions are worsening [1, 5, 6, 7]. For example, in England in 2024, 85% of adults who smoked inaccurately perceived that vaping is equally or more harmful than smoking or did not know the relative harms, an increase from 59% in 2014 [7]. These misperceptions can deter people who smoke from switching to vaping and quitting smoking [1, 2]. Interventions are therefore required to address these misperceptions.

Misleading news, especially around incidences of acute lung injuries from vaping contaminated cannabis e‐cigarettes (predominantly in the USA), has worsened public perceptions of vaping in the UK and elsewhere [8]. Claims on social media that have exaggerated the health risks of vaping have also been found to further exacerbate vaping misperceptions [9]. Misunderstanding of the health harms of nicotine may also contribute to inaccurate perceptions of vaping [1, 10]. Effectively communicating comparative risk information for e‐cigarettes and combusted cigarettes is therefore needed.

Evidence reviews have suggested that interventions communicating that vaping is less harmful than smoking are effective in improving this perception [1, 2, 11]. One study among UK adults who smoke found that academic expert videos improved harm perceptions and increased intentions to try vaping, and that videos outperformed text‐based messages [12]. However, to our knowledge there have been no studies assessing interventions to change misperceptions among young adults in the UK [1, 2, 11]. Young adults are an important group to research because they have the highest rates of experimenting with smoking [13], which may lead to long‐term continued smoking [14], and would also benefit greatly from switching to vaping because the earlier someone quits smoking the better their health outcomes [15].

Social media dissemination of brief video clips offers advantages of timely and broad reach for interventions to correct vaping misperceptions among young adults [16, 17, 18, 19, 20]. Source credibility also has an important impact on the trust and effectiveness of health risk messages, particularly for smoking [21] and vaping [22]. Therefore, videos led by academic experts discussing research evidence may offer additional benefits beyond more generic videos [23, 24, 25, 26, 27].

In this study, we therefore evaluate the impact of a series of eight vaping facts videos, designed for social media, in which academic experts discuss the evidence on vaping and respond to common misconceptions regarding vaping and smoking. The aim of this study is to evaluate whether vaping facts videos can change vaping harm perceptions among young adults in the UK. We hypothesized that exposure to the vaping facts videos will lead to an increase in the accurate perception that vaping is less harmful than smoking compared with the control group. We also assessed the effectiveness of each individual video as well as changes in perceptions that vaping is harmful and addictive, changes in common misconceptions, and subgroup differences by smoking and vaping status.

## METHODS

### Pre‐registration

This study was pre‐registered on the Open Science Framework and the protocol and survey are available on‐line (https://osf.io/ujsf8) [28].

### Design

This was an on‐line between‐subjects experimental study conducted on Qualtrics, a web‐based survey platform, in May 2024. Participants were randomized to one of two conditions (experimental vs control) in a 1 : 1 allocation ratio. Those in the experimental group were further randomized to view one of eight brief videos, each targeting a different vaping‐related misconception. This allowed for assessment of both overall and perception‐specific effects. Before and after exposure to the videos, all participants answered questions about their perceptions of vaping and smoking (Figure S1).

### Participants

**FIGURE 1:**
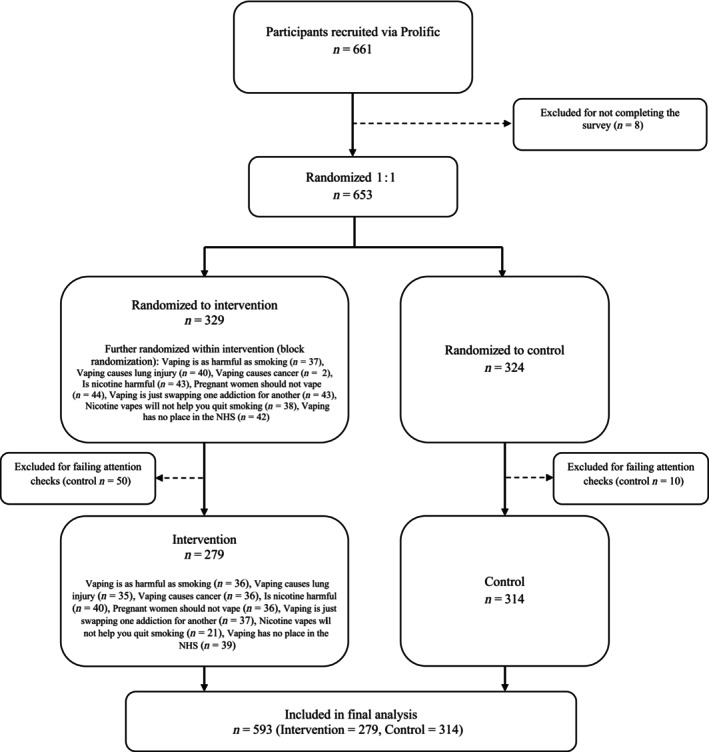
CONSORT participant flow diagram.

Participants were recruited via Prolific Academic. Participants were eligible to take part if they were aged between 18 and 30 years, currently resided in the UK and passed the attention check embedded in the survey. Participants received a reimbursement of £0.90 upon completion, in line with Prolific Academic’s incentive structure. An equal number of male and female participants, and numbers per each smoking/vaping subgroup, were recruited. The attention check involved asking participants ‘Who was the speaker in the video you just watched?’, followed by a list of academic experts and with the correct answer based on the speaker in the participant’s assigned video. The attention check was designed to ensure that participants engaged with the video content and is a common quality control measure used in on‐line experiments [29]. This approach is consistent with best practice in on‐line studies, where attention checks are widely recommended to exclude inattentive respondents. A participant flow diagram is shown in Figure 1.

### Procedure

Participants were recruited through Prolific and provided informed consent via an on‐line information sheet. After completing demographic, smoking/vaping status and baseline perception questions, and viewing the video, participants completed an attention check. All outcome measures were collected immediately post‐exposure. Participants were not informed which condition they were in, nor were they aware of the presence of eight different videos. All participants received the same outcome measures in the same order, regardless of condition. No investigators were involved in the allocation process, and no personal identifiers were collected at any stage. All videos began by displaying the speaker’s name, institutional affiliation and the title for the first 5 seconds, and most also verbally introduced the speaker during this time.

### Randomization

Randomization was stratified by sex (male/female) and smoking/vaping subgroup (exclusive smokers, exclusive vapers, dual users and non‐users) to ensure balanced allocation across conditions. Within the intervention condition, participants were further randomized to one of eight expert‐led videos, each addressing a different vaping‐related misconception. This second randomization was also conducted automatically within Qualtrics using block randomization.

### Interventions

#### Experimental condition

**FIGURE 2:**
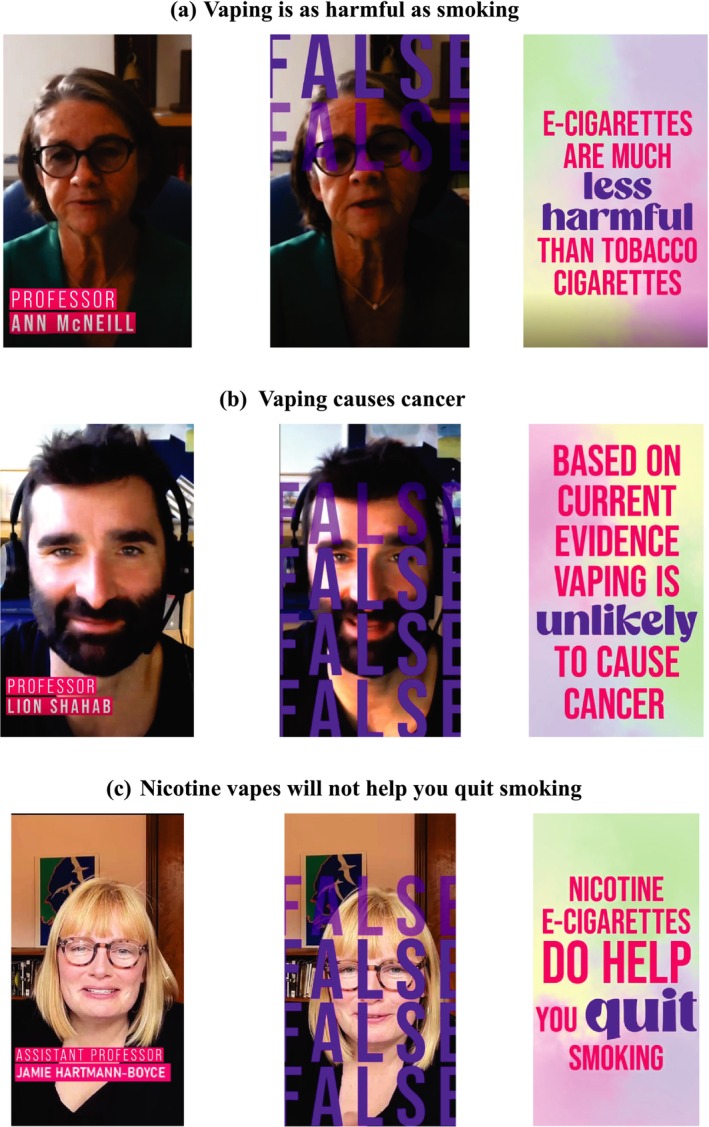
Examples of the videos shown in the experimental condition.Examples of the videos shown in the experimental condition.

Participants were randomized to view one of eight vaping facts videos developed by the University of East Anglia and the Office for Health Improvement and Disparities (https://www.youtube.com/playlist?list=PLgQTgYsPq_80WoIkB5QjRPAz2H_TEwY49). The videos involved academics discussing the evidence on the harms of vaping and/or vaping for quitting smoking with the Tobacco Control Lead of the Office for Health Improvement and Disparities. Hide and Seek Media produced the videos. Each video was approximately 1 minute in duration. In this study, the video titles were set up as ‘misconceptions’ or commonly heard statements that the videos set out to address: (i) vaping is as harmful as smoking (*n* = 36); (ii) vaping causes lung injury (*n* = 35); (iii) vaping causes cancer (*n* = 36); (iv) nicotine is harmful (*n* = 40); (v) Pregnant women should not vape (*n* = 36); (vi) vaping is just swapping one addiction for another (*n* = 37); (vii) nicotine vapes will not help you quit smoking (*n* = 20); (viii) vaping has no place in the UK National Health Service (NHS) (*n* = 39). Videos presented nuanced information based on the scientific literature at the time of the research [1, 10, 12]. Each video was designed to influence the primary outcome – whether participants perceive vaping as less harmful than smoking – by correcting false beliefs about the health effects of vaping or its role in smoking cessation. For example, the video ‘nicotine vapes will not help you quit smoking’ addressed misperceptions about the effectiveness of vaping as a cessation aid, thereby supporting the broader message that vaping is less harmful than smoking. Figure 2 displays some examples of the videos shown and the supporting information provides transcripts and stills from the videos (Figure S2).

#### Control condition

Participants viewed a 1‐minute video about pancake facts produced by King’s College London (https://www.youtube.com/shorts/YqOh7-niVdc?si=dyOYejNmTOb9i2iJ).

### Measures

#### Primary outcome

Perception that vaping is less harmful than smoking. ‘Is using e‐cigarettes/vaping less harmful, about the same or more harmful than smoking cigarettes?’, coded as less harmful (selecting either ‘a little less harmful than “regular” tobacco cigarettes’ or ‘a lot less harmful than “regular” tobacco cigarettes’) versus otherwise (‘a lot more harmful than “regular” tobacco cigarettes’, ‘a little more harmful than “regular” tobacco cigarettes’, ‘as harmful as “regular” tobacco cigarettes’ or ‘do not know’).

#### Secondary outcomes

Perception that vaping is not at all harmful. ‘How harmful do you think it is to use e‐cigarettes/vape?’, coded as ‘not at all harmful’ versus otherwise (‘slightly harmful’, ‘somewhat harmful’, ‘very harmful’, ‘extremely harmful’ or ‘do not know’).

Perception that vaping is not at all addictive. ‘How addictive are e‐cigarettes/vaping?’, coded as ‘not at all addictive’ versus otherwise (‘slightly addictive’, ‘somewhat addictive’, ‘extremely addictive’ or ‘do not know’).

Misconceptions. Participants were also presented with a series of statements that were matched to the misconceptions addressed in the videos. These statements reflect misconceptions addressed in the videos and represent current evidence for nicotine vaping of licit UK products at the time of the study [1, 10, 12]. Response options were ‘true’, ‘false’ or ‘do not know’ for each: (i) vaping is as harmful as smoking (correct answer: false); (ii) vaping causes lung injury (correct answer: false); (iii) vaping causes cancer (correct answer: false); (iv) when used in ways that does not involve smoking tobacco, nicotine is harmful (correct answer: false); (v) pregnant women should not vape (correct answer: false); (vi) vaping is just swapping one addiction for another (correct answer: true); (vii) nicotine vapes will not help you quit smoking (correct answer: false); (viii) vaping has no place in the NHS (correct answer: false). Response options were coded as ‘correct’ or ‘otherwise’ (i.e. incorrect response or do not know).

Not curious about vaping. Not curious about vaping, among those who had never vaped only. ‘Are you curious about using e‐cigarettes?’, coded as ‘definitely not’ versus otherwise (‘probably not’, ‘probably yes’, ‘definitely yes’ or ‘do not know’).

No intention to try vaping. No intention to try vaping, among those who had never vaped only. ‘Do you think that you will use e‐cigarettes/vape in the next 12 months?’, coded as ‘definitely not’ versus otherwise (‘probably not’, ‘probably yes’, ‘definitely yes’ or ‘do not know’).

#### Baseline/background characteristics

Age. Ages 18–20 years, 21–24 years or 25–30 years.

Sex. Female, male and prefer not to say.

Education. Below sixth form, sixth form, university degree, higher than university degree or other.

Ethnicity. White, mixed/multiple ethnic groups, Asian/Asian British, Black/Black British/Caribbean/African, prefer not to say or other.

Social media platform usage. Use any platform listed (Facebook, Instagram, TikTok, X/Twitter, Snapchat) or otherwise (none selected or do not know).

Past 30‐day vaping/smoking. Both vaped and smoked in the past 30 days, only vaped in the past 30 days, only smoked in the past 30 days or neither. Details on how these were derived are available on‐line (https://osf.io/ujsf8).

### Statistical analysis

A pre‐planned sample size calculation (https://osf.io/ujsf8) [28] indicated that we needed 520 participants (260 per group) to observe a small effect size (*d* = 0.2, OR = 1.44) with 95% power and an alpha level of 5%. Accounting for an anticipated 80% retention/inclusion rate, we recruited a total of 661 participants (331 in the experimental group and 330 in the control group). Of the 661 that were recruited, 60 were excluded for failing the attention check, and eight were further excluded for not completing the survey, leaving 593 in the analytic sample.

Descriptive statistics were employed by calculating frequencies and percentages for categorical variables.

First, unadjusted and adjusted (for age group, education, ethnicity, use of social media, vaping/smoking status) logistic regression models were fitted for each outcome to examine whether condition (experimental vs control) was associated with each outcome. Second, logistic regression analyses were conducted separately for each of the eight video conditions, to assess the individual impact of each video on outcomes (additional to pre‐registration). Third, two‐way interactions between condition and vaping/smoking status were added to the logistic regression models, to test for a differential condition effect according to the participant’s vaping/smoking status. Owing to small sample sizes, interactions were not tested for the logistic regressions stratified by video condition. There were no missing data from the analytic sample because participants who failed the attention check (*n* = 60) or who did not complete the survey (*n* = 8) were excluded prior to analyses.

All analyses were conducted using SPSS Statistics 26 (IBM, Armonk, NY, USA). Descriptive statistics, crosstabulations and binary logistic regression models were used to assess the main effects and subgroup differences.

### Ethics

Approval for this research was granted by the Research Ethics Office at King’s College London (approval code: MRA‐23/24‐41237). Participants provided consent electronically.

## RESULTS

### Sample characteristics (Table 1)

**TABLE 1:** Sample characteristics and differences between those randomized to the intervention and those randomized to the control conditions.

	Total (*n* = 593)	Intervention (*n* = 279)	Control (*n* = 314)
	% (*n*)	% (*n*)	% (*n*)
**Age group**			
18–20 years	3.7 (22)	3.6 (10)	3.8 (12)
21–24 years	29.5 (175)	31.5 (88)	27.7 (87)
25–30 years	66.8 (396)	64.9 (181)	68.5 (215)
**Sex**			
Female	49.7 (295)	51.3 (143)	48.4 (152)
Male	49.2 (292)	47.7 (133)	50.6 (159)
Prefer not to say	1.0 (6)	1.1 (3)	1 (3)
**Education**			
Below sixth form	7.4 (44)	8.2 (23)	6.7 (21)
Sixth form	62.9 (373)	60.6 (169)	65 (204)
University degree	28.3 (168)	29.4 (82)	27.4 (86)
Higher than university degree	1.0 (6)	1.8 (5)	0.3 (1)
Other	0.3 (2)	0 (0)	0.6 (2)
**Ethnicity**			
White	76.1 (451)	77.4 (216)	74.8 (235)
Mixed/multiple ethnic groups	6.6 (39)	6.8 (19)	6.4 (20)
Asian/Asian British	10.6 (63)	10.4 (29)	10.8 (34)
Black/Black British/Caribbean/African	4.9 (29)	3.9 (11)	5.7 (18)
Prefer not to say	0.8 (5)	0.7 (2)	1 (3)
Other	1.0 (6)	0.7 (2)	1.3 (4)
**Social media**			
Use any platform	98.0 (581)	98.2 (274)	97.8 (307)
Otherwise	2.0 (12)	1.8 (5)	2.2 (7)
**Vaping is less harmful than smoking (pre‐intervention)**			
Otherwise	39 (231)	39.4 (110)	38.5 (121)
Less harmful	61 (362)	60.6 (169)	61.5 (193)
**Vaping is not at all harmful (pre‐intervention)**			
Otherwise	99.2 (588)	100 (279)	98.4 (309)
Not at all harmful	0.8 (5)	0 (0)	1.6 (5)
**Vaping is not at all addictive (pre‐intervention)**			
Otherwise	98.5 (584)	98.9 (276)	98.1 (308)
Not at all addictive	1.5 (9)	1.1 (3)	1.9 (3)
**Vaping/smoking status**			
Both vaped and smoked	28.7 (170)	28.3 (79)	29 (91)
Only vaped	32 (190)	29.4 (82)	34.4 (108)
Only smoked	8.9 (53)	11.1 (31)	7 (22)
Neither vaped nor smoked	30.4 (180)	31.2 (87)	29.6 (93)

The majority of participants were aged 25–30 years, had completed sixth form or higher education, identified as white race/ethnicity and had used social media. There was an equal proportion of males and females. At baseline, most (61.0%) perceived vaping as less harmful than smoking, while few perceived that vaping is not at all harmful (0.8%) or not at all addictive (1.5%).

### Associations between video intervention and vaping perceptions and intentions (Table 2)

**TABLE 2:** Logistic regression assessing differences in perceptions and intentions by video condition.

		*n* (%)	Unadjusted		Adjusted^a^	
			OR (95% CI)	*P*	OR (95% CI)	*P*
**Vaping perceptions (*n* = 593)**						
Vaping is less harmful than smoking	Control (ref.)	181 (57.6%)	1.00		1.00	
	Intervention	229 (82.1%)	**3.37 (2.30–4.92)**	**<0.001**	**3.69 (2.49–5.47)**	**<0.001**
Vaping is not harmful	Control (ref.)	5 (1.6%)	1.00		1.00	
	Intervention	9 (3.2%)	2.06 (0.68–6.22)	0.200	2.57 (0.78–8.52)	0.122
Vaping is not addictive	Control (ref.)	3 (1.0%)	1.00		1.00	
	Intervention	2 (0.7%)	0.75 (0.12–4.51)	0.752	0.49 (0.04–6.67)	0.594
**Knowledge checks (*n* = 593)**						
Vaping is as harmful as smoking – false	Control (ref.)	113 (36%)	1.00		1.00	
	Intervention	190 (68.1%)	**3.80 (2.70–5.34)**	**<0.001**	**4.14 (2.90–5.90)**	**<0.001**
Vaping causes lung injury – false	Control (ref.)	15 (4.8%)	1.00		1.00	
	Intervention	58 (20.8%)	**5.23 (2.89–9.47)**	**<0.001**	**6.08 (3.28–11.27)**	**<0.001**
Vaping causes cancer – false	Control (ref.)	42 (13.4%)	1.00		1.00	
	Intervention	86 (30.8%)	**2.89 (1.91–4.36)**	**<0.001**	**3.17 (2.07–4.84)**	**<0.001**
When used in ways that does not involve smoking tobacco, nicotine is harmful – false	Control (ref.)	57 (18.2%)	1.00		1.00	
	Intervention	89 (31.9%)	**2.11 (1.44–3.09)**	**<0.001**	**2.21 (1.49–3.29)**	**<0.001**
Pregnant women should not vape – false	Control (ref.)	5 (1.6%)	1.00		1.00	
	Intervention	28 (10.0%)	**6.89 (2.62–18.11)**	**<0.001**	**8.79 (3.26–23.70)**	**<0.001**
Vaping is just swapping one addiction for another – true	Control (ref.)	275 (87.6%)	1.00		1.00	
	Intervention	230 (82.4%)	0.67 (0.42–1.05)	0.080	0.62 (0.39–1.00)	0.050
Nicotine vapes will not help you quit smoking – false	Control (ref.)	110 (35.0%)	1.00		1.00	
	Intervention	142 (50.9%)	**1.92 (1.38–2.67)**	**<0.001**	**1.99 (1.42–2.81)**	**<0.001**
Vaping has no place in the NHS – false	Control (ref.)	85 (27.1%)	1.00		1.00	
	Intervention	101 (36.2%)	**1.53 (1.08–2.17)**	**0.** **017**	**1.57 (1.09–2.25)**	**0.** **015**
**Curiosity and intention to vape (never vapers, *n* = 54)**						
Not curious about trying vaping	Control (ref.)	30 (90.9%)	1.00		1.00	
	Intervention	18 (85.7%)	0.60 (0.12–3.30)	0.557	0.55 (0.06–5.06)	0.594
No intention to try vaping	Control (ref.)	32 (97.0%)	1.00		1.00	
	Intervention	18 (85.7%)	0.19 (0.02–1.94)	0.160	–^b^	–

*Note*: Bolded figures show associations with *P* < 0.05.

Abbreviation: NHS = UK National Health Service.

^a^ Analyses were adjusted for age group, education, ethnicity, use of social media and vaping/smoking status.

^b^ Owing to insufficient cell sizes when adjusting for covariates (i.e. some *n* = 0), regression cannot be run.

As hypothesized [28], after exposure to an intervention video, the accurate perception that vaping is less harmful than smoking was greater than after exposure to the control video. There was little evidence for an effect of the intervention (vs control) on perceptions that vaping is not harmful or not addictive. Adjusted and unadjusted results were very similar in direction and magnitude (Table 2).

For knowledge checks, except the perception that vaping is just swapping one addiction for another, correctly identifying all other statements as false were higher among those who had viewed an intervention video compared with the control (Table 2).

Among participants who had never vaped, there was little difference in curiosity or intention to vape between those exposed to intervention or control videos. However, sample sizes for these associations were small, limiting confidence in the findings, and adjusted analyses could not be run for intention to try vaping because of insufficient sample sizes in the model when adjusting for covariates.

### Associations between the video intervention and vaping perceptions and intentions, stratified by video (Table 3)

**TABLE 3:** Logistic regression analyses assessing differences in vaping perceptions, stratified by video (*n* = 593).

Perception (outcome) stratified by video (exposure)	*n* (%)	Unadjusted		Adjusted^a^	
		OR (95% CI)	P	OR (95% CI)	P
**Vaping is less harmful than smoking – true**					
Control video (ref.)	181 (57.6%)	1.00		1.00	
Vaping is as harmful as smoking video	34 (94.4%)	**12.49 (2.95–52.91)**	**<0.001**	**13.92 (3.26–59.37)**	**<0.001**
Vaping causes lung injury video	32 (91.4%)	**7.84 (2.35–26.14)**	**<0.001**	**8.45 (2.49–28.68)**	**<0.001**
Vaping causes cancer video	32 (88.9%)	**5.88 (2.03–17.02)**	**0.001**	**5.95 (2.02–17.5)**	**0.** **001**
Nicotine is harmful video	28 (70%)	1.72 (0.84–3.50)	0.138	1.77 (0.83–3.75)	0.138
Pregnant women should not vape video	33 (91.7%)	**8.08 (2.43–26.19)**	**<0.001**	**9.14 (2.69–31.03)**	**<0.001**
Vaping is just swapping one addiction for another video	32 (86.5%)	**4.70 (1.79–12.39)**	**0.002**	**5.37 (1.99–14.43)**	**<0.001**
Nicotine vapes will not help you quit smoking video	16 (80.0%)	2.94 (0.96–8.99)	0.059	**3.45 (1.06–11.21)**	**0.** **039**
Vaping has no place in the NHS video	22 (56.4%)	0.95 (0.49–1.86)	0.883	0.94 (0.46–1.89)	0.850
**Vaping is as harmful as smoking – false**					
Control video (ref.)	113 (36.0%)	1.00		1.00	
Vaping is as harmful as smoking video	32 (88.9%)	**14.23 (4.91–41.27)**	**<0.001**	**16.56 (5.64–48.67)**	**<0.001**
Vaping causes lung injury video	30 (85.7%)	**10.67 (4.03–28.28)**	**<0.001**	**11.69 (4.33–31.53)**	**<0.001**
Vaping causes cancer video	29 (80.6%)	**7.37 (3.13–17.36)**	**<0.001**	**7.29 (3.05–17.35)**	**<0.001**
Nicotine is harmful video	22 (55%)	**2.17 (1.12–4.22)**	**0.** **022**	**2.31 (1.15–4.66)**	**0.** **019**
Pregnant women should not vape video	26 (72.2%)	**4.63 (2.15–9.94)**	**<0.001**	**4.98 (2.28–10.88)**	**<0.001**
Vaping is just swapping one addiction for another video	23 (62.2%)	**2.92 (1.45–5.90)**	0.**003**	**3.16 (1.54–6.51)**	0.**002**
Nicotine vapes will not help you quit smoking video	13 (65.0%)	**3.30 (1.28–8.52)**	0.**013**	**3.45 (1.30–9.16)**	0.**013**
Vaping has no place in the NHS video	15 (38.5%)	1.11 (0.56–2.21)	0.762	1.16 (0.58–2.36)	0.672
**Vaping causes lung injury – false**					
Control video (ref.)	15 (4.8%)	1.00		1.00	
Vaping is as harmful as smoking video	5 (13.9%)	**3.22 (1.09–9.44)**	**0.** **034**	**3.59 (1.17–11.03)**	**0.** **026**
Vaping causes lung injury video	30 (85.7%)	**119.60 (40.64–352)**	**<0.001**	**177.85 (55.08–574.34)**	**<0.001**
Vaping causes cancer video	8 (22.2%)	**5.69 (2.22–14.60)**	**<0.001**	**7.75 (2.83–21.23)**	**<0.001**
Nicotine is harmful video	7 (17.5%)	**4.23 (1.61–11.12)**	**0.** **003**	**6.21 (2.21–17.40)**	**<0.001**
Pregnant women should not vape video	3 (8.3%)	1.81 (0.50–6.59)	0.367	1.90 (0.50–7.18)	0.345
Vaping is just swapping one addiction for another video	2 (5.4%)	1.14 (0.25–5.19)	0.866	1.07 (0.23–5.08)	0.929
Nicotine vapes will not help you quit smoking video	2 (10.0%)	2.22 (0.47–10.44)	0.315	2.88 (0.56–14.73)	0.204
Vaping has no place in the NHS video	1 (2.6%)	0.53 (0.07–4.08)	0.538	0.60 (0.07–4.94)	0.631
**Vaping causes cancer – false**					
Control video (ref.)	42 (13.4%)	1.00		1.00	
Vaping is as harmful as smoking video	7 (19.4%)	1.56 (0.64–3.80)	0.324	1.56 (0.63–3.84)	0.335
Vaping causes lung injury video	16 (45.7%)	**5.45 (2.60–11.43)**	**<0.001**	**5.65 (2.64–12.10)**	**<0.001**
Vaping causes cancer video	32 (88.9%)	**51.81 (17.44–153.95)**	**<0.001**	**60.36 (19.70–184.88)**	**<0.001**
Nicotine is harmful video	9 (22.5%)	1.88 (0.84–4.23)	0.127	**2.33 (1.00–5.41)**	**0.** **049**
Pregnant women should not vape video	7 (19.4%)	1.56 (0.64–3.80)	0.324	1.65 (0.66–4.09)	0.282
Vaping is just swapping one addiction for another video	9 (24.3%)	2.08 (0.92–4.72)	0.079	2.22 (0.96–5.14)	0.063
Nicotine vapes will not help you quit smoking video	3 (15.0%)	1.14 (0.32–4.07)	0.837	1.34 (0.36–4.97)	0.667
Vaping has no place in the NHS video	3 (7.7%)	0.54 (0.16–1.83)	0.322	0.61 (0.18–2.10)	0.432
**When used in ways that does not involve smoking tobacco, nicotine is harmful – false**					
Control video (ref.)	57 (18.2%)	1.00		1.00	
Vaping is as harmful as smoking video	9 (25.0%)	1.50 (0.67–3.37)	0.322	1.60 (0.70–3.67)	0.266
Vaping causes lung injury video	13 (37.1%)	**2.66 (1.27–5.60)**	**0.** **010**	**2.97 (1.38–6.39)**	**0.** **005**
Vaping causes cancer video	6 (16.7%)	0.90 (0.36–2.27)	0.826	0.98 (0.38–2.54)	0.968
Nicotine is harmful video	26 (65.0%)	**8.37 (4.12–17.04)**	**<0.001**	**9.73 (4.57–20.68)**	**<0.001**
Pregnant women should not vape video	12 (33.3%)	**2.25 (1.07–4.77)**	**0.** **034**	**2.53 (1.16–5.50)**	**0.** **019**
Vaping is just swapping one addiction for another video	8 (21.6%)	1.24 (0.54–2.86)	0.608	1.25 (0.53–2.92)	0.613
Nicotine vapes will not help you quit smoking video	7 (35.0%)	2.43 (0.93–6.36)	0.071	2.42 (0.87–6.72)	0.089
Vaping has no place in the NHS video	8 (20.5%)	1.16 (0.51–2.66)	0.720	1.17 (0.50–2.77)	0.718
**Vaping is just swapping one addiction to another – true**					
Control video (ref.)	275 (87.6%)	1.00		1.00	
Vaping is as harmful as smoking video	28 (77.8%)	0.50 (0.21–1.17)	0.108	0.42 (0.18–1.03)	0.057
Vaping causes lung injury video	26 (74.3%)	**0.41 (0.18–0.94)**	**0.** **035**	**0.39 (0.16–0.91)**	**0.** **029**
Vaping causes cancer video	31 (86.1%)	0.88 (0.32–2.40)	0.801	0.75 (0.27–2.11)	0.590
Nicotine is harmful video	35 (87.5%)	0.99 (0.37–2.69)	0.989	0.94 (0.33–2.69)	0.902
Pregnant women should not vape video	29 (80.6%)	0.59 (0.24–1.43)	0.242	0.59 (0.23–1.47)	0.254
Vaping is just swapping one addiction for another video	32 (86.5%)	0.91 (0.33–2.47)	0.849	0.97 (0.34–2.71)	0.948
Nicotine vapes will not help you quit smoking video	15 (75%)	0.43 (0.15–1.24)	0.116	0.40 (0.13–1.23)	0.108
Vaping has no place in the NHS video	34 (87.2%)	0.96 (0.36–2.61)	0.943	0.92 (0.32–2.66)	0.874
**Nicotine vapes will not help you quit smoking – false**					
Control video (ref.)	110 (35.0%)	1.00		1.00	
Vaping is as harmful as smoking video	23 (63.9%)	**3.28 (1.60–6.73)**	**0.** **001**	**3.79 (1.81–7.95)**	**<0.001**
Vaping causes lung injury video	16 (45.7%)	1.56 (0.77–3.16)	0.215	1.64 (0.79–3.39)	0.185
Vaping causes cancer video	17 (47.2%)	1.66 (0.83–3.32)	0.153	1.68 (0.82–3.45)	0.155
Nicotine is harmful video	19 (47.5%)	1.68 (0.87–3.25)	0.126	1.70 (0.84–3.42)	0.138
Pregnant women should not vape video	17 (47.2%)	1.66 (0.83–3.32)	0.153	1.76 (0.86–3.62)	0.122
Vaping is just swapping one addiction for another video	17 (45.9%)	1.58 (0.79–3.13)	0.194	1.65 (0.81–3.35)	0.166
Nicotine vapes will not help you quit smoking video	16 (80.0%)	**7.42 (2.42–22.73)**	**<0.001**	**7.09 (2.25–22.36)**	**<0.001**
Vaping has no place in the NHS video	17 (43.6%)	1.43 (0.73–2.81)	0.295	1.44 (0.72–2.90)	0.304
**Vaping has no place on the NHS – false**					
Control video (ref.)	85 (27.1%)	1.00		1.00	
Vaping is as harmful as smoking video	13 (36.1%)	1.52 (0.74–3.14)	0.255	1.69 (0.80–3.56)	0.166
Vaping causes lung injury video	12 (34.3%)	1.41 (0.67–2.95)	0.368	1.48 (0.69–3.18)	0.313
Vaping causes cancer video	13 (36.1%)	1.52 (0.74–3.14)	0.255	1.58 (0.74–3.35)	0.234
Nicotine is harmful video	13 (32.5%)	1.30 (0.64–2.63)	0.471	1.24 (0.59–2.62)	0.568
Pregnant women should not vape video	14 (38.9%)	1.71 (0.84–3.50)	0.139	1.89 (0.90–3.94)	0.091
Vaping is just swapping one addiction for another video	10 (27.0%)	1.00 (0.46–2.15)	0.996	1.03 (0.47–2.26)	0.942
Nicotine vapes will not help you quit smoking video	10 (50.0%)	**2.69 (1.08–6.70)**	**0.** **033**	2.51 (0.96–6.54)	0.060
Vaping has no place in the NHS video	16 (41.0%)	1.87 (0.95–3.72)	0.072	1.82 (0.90–3.70)	0.098

*Note*: Bolded figures show associations with *P* < 0.05.

Abbreviation: NHS = National Health Service.

Owing to insufficient sample sizes, regressions with perceptions that vaping is not harmful, vaping is not addictive, and curiosity and intention to vape were not run.

^a^ Analyses were adjusted for age group, education, ethnicity, use of social media and vaping/smoking status.

Overall, perceptions and knowledge were most accurate among participants who had viewed the most relevant expert video. For example, 94.4% of participants exposed to the video debunking the misconception ‘vaping is as harmful as smoking’ accurately perceived vaping as less harmful than smoking, compared to 57.6% exposed to the control video. A spillover effect was also observed, whereby misconception‐specific videos also influenced other perceptions. For example, exposure to the video debunking the misconception that vaping causes lung injury increased the proportion of respondents who responded ‘false’ to the statements ‘vaping is as harmful as smoking’, ‘vaping causes cancer’ and ‘when used in ways that does not involve smoking tobacco, nicotine is harmful’.

Owing to the small numbers of participants who selected the following outcomes, regression analyses stratified by video could not be conducted: perceptions that vaping is not harmful (*n* = 14), vaping is not addictive (*n* = 5), and curiosity (*n* = 48) and intention to vape (*n* = 50).

### Interactions between vaping/smoking status and the video intervention

There was little evidence of any interactions between condition and vaping/smoking status for any outcomes (all *P* > 0.05; Table S1). This suggests that the videos had a consistent effect among those who both vaped and smoked, only vaped, only smoked or did neither. However, the sample sizes were too small to assess interactions for the perception that vaping is not harmful, vaping is not addictive or that pregnant people should not vape.

## DISCUSSION

This study assessed whether brief, academic‐led, evidence‐based social media videos about vaping were effective in correcting young adults’ perceptions of vaping. The findings suggest that, overall, the videos were effective in improving accurate vaping perceptions and debunking common misconceptions about vaping. As hypothesized [28], post‐intervention, participants who had viewed an expert video had three times the odds of accurately perceiving vaping as less harmful than smoking compared with participants who had viewed the control video. They also more commonly rejected several misconceptions about vaping when compared with those who viewed the control group video. Perceptions that vaping is harmful and addictive remained high, even after the intervention videos. Further, some beliefs, such as the idea that pregnant people should not vape, also showed lower overall endorsement, even among the intervention group. These beliefs may be more resistant to change because of stronger pre‐existing attitudes, heightened concern about pregnancy‐related risks or ambiguity in interpreting public health messages.

Findings are consistent with prior research findings that interventions communicating that vaping is less harmful than smoking can increase this perception [1, 2, 12]. The one video specifically focusing on debunking the misconception that vaping is as harmful as smoking had the greatest effect on increasing the accurate perception that vaping is less harmful than smoking. Such a targeted approach clearly demonstrates that it is possible to correct young people’s misperceptions of vaping using brief expert information, at least in the short term.

There were also positive effects of the informational videos on improving knowledge about vaping more widely than relative harm perceptions. For instance, the inaccurate beliefs that vaping causes lung injury, leads to cancer or when used in ways that does not involve smoking tobacco, nicotine is harmful, were all less prevalent in the intervention video groups. This is again consistent with previous studies [12].

Our study found some spillover effects, demonstrating that watching videos aimed at dispelling certain misconceptions also influenced beliefs for other misconceptions. For example, those who viewed the video debunking the misconception that ‘Vaping causes lung injury’ were more likely to reject the statement ‘When used in ways that does not involve smoking tobacco, nicotine is harmful’, compared with those in the control condition. Therefore, videos debunking common misconceptions could have a wider impact on many different misperceptions. There may be concern that interventions to tackle vaping misperceptions could have spillover effects that could promote smoking behaviour; however, previous research of US campaigns found that anti‐vaping campaigns did not affect attitudes towards smoking [30].

While many perceptions were improved after exposure to the academic‐led videos, the majority of young adults continued to perceive vaping as addictive (99.3%) and harmful (96.8%). While less harmful than smoking, the long‐term health harms of vaping are unknown and research suggests that vaping exposes users to some harmful toxicants [1, 3, 31]. Vaping has the potential to be addictive and markers of dependence are increasing among young people [32]. Therefore, it is promising that the videos did not significantly impact these perceptions.

This study also sought to assess whether curiosity and intention to vape were affected by the academic‐led videos; however, the sample sizes were too small to enable confident comparisons. Prior research has found that comparative risk messages increased intentions to vape among adults who smoke [22, 33], but also that perceiving vaping as harmful decreases intentions to vape among young people [34]. The present study would therefore benefit from replication with larger numbers of young adults who did not vape, to understand the specific impact on this group. However, it is positive that the majority of the sample who had never vaped were not curious about or intended to try vaping, even after exposure to the academic‐led videos.

This study has some important limitations. First, a forced‐exposure paradigm was used, which lacks ecological validity, thereby limiting the conclusions we can make about the effectiveness of these videos outside our experimental setting. Second, the sample was not nationally representative and the people who join on‐line survey panels such as Prolific are unlikely to represent the UK population. There was an over‐representation of participants who were of white race/ethnicity, and more participants had accurate harm perceptions of vaping than the general population of adults [6]. The findings are therefore not necessarily generalizable to the population of young adults in the UK or other countries. Third, only short‐term shifts in perceptions and intended behaviours were captured, and it is unclear whether these were sustained in the long term or translated to changes in actual behaviours. Fourth, as described above, the sample sizes were too small to assess changes in some outcomes (e.g. vaping is addictive, curiosity about vaping) and, when running the analyses for each video, sample sizes were low and confidence intervals were wide, thus contributing to uncertainty in estimates. Future research should replicate this study using larger, nationally representative samples and incorporate follow‐up evaluations to determine the longer‐term impacts of the intervention on perceptions and behaviour change. Fifth, although the videos and associated statements used to assess vaping knowledge reflected current evidence at the time of the study, some may oversimplify complex scientific issues. Findings from these outcomes should therefore be interpreted with caution.

The strengths of the study include, first, the randomized design, allowing for more confident conclusions to be made about the effects of the videos on perceptions. Second, several vaping perceptions and misconceptions were assessed to ensure an overall evaluation of the effectiveness of the intervention, with some measures being more specific to the videos that were shown than others. Prior work has highlighted the importance in considering a range of outcomes when evaluating health interventions [35]. Third, the videos involved credible sources (government and academic experts), which has been found to increase the effectiveness of smoking and vaping interventions [21, 22]. Fourth, measures were taken to increase data quality, such as incorporating attention checks and removing those who failed.

## CONCLUSION

This study was the first to evaluate the impact of short academic‐led, evidence‐based videos designed for social media on correcting vaping misperceptions among young adults in the UK. It found that the videos were effective in correcting vaping misperceptions, including the common misperception that vaping is as, or more, harmful than smoking. The findings are timely owing to escalating misperceptions in the UK.

## AUTHOR CONTRIBUTIONS

**Mohammad Alharbi:** Conceptualization (equal); data curation (lead); formal analysis (lead); investigation (equal); methodology (equal); writing—original draft (lead). **Emma Ward:** Conceptualization (equal); funding acquisition (equal); investigation (equal); methodology (equal); project administration (equal); resources (lead); writing—review and editing (equal). **Caitlin Notley:** Conceptualization (equal); funding acquisition (lead); investigation (equal); methodology (equal); resources (lead); writing—review and editing (equal). **Martin Dockrell:** Conceptualization (equal); investigation (equal); resources (equal); writing—review and editing (equal). **Eve Taylor:** Investigation (supporting); writing—review and editing (equal). **Katherine East:** Conceptualization (equal); data curation (supporting); formal analysis (supporting); funding acquisition (equal); investigation (equal); methodology (equal); project administration (equal); resources (equal); supervision (lead); validation (equal); writing—original draft (supporting).

## DECLARATION OF INTERESTS

At the time of this study, M.D. was the Tobacco Control Programme Lead at the Office for Health Improvement and Disparities (OHID). The videos were developed by the University of East Anglia (UEA). C.N. (UEA) featured in one of the videos evaluated in this study. K.E. (KCL) featured in one video but this was not evaluated as part of this study. M.D. was the lead interviewer in the videos.

## Supporting information


**Figure S1.** Study design and details on when measures were collected.
**Figure S2.** Videos shown in the experimental condition.
**Table S1.** Interactions between vaping/smoking status and intervention in predicting vaping harm perceptions, adjusting for covariates.

## Data Availability

The data and code are available on the Open Science Framework: https://osf.io/ckmys/.
