# Supplementary material for: Evaluating the impact of vaping facts films on vaping harm perceptions among young adults in the UK: A randomized on‐line experiment
Source: Addiction. 2025 Jul 9;120(11):2202–14. doi: 10.1111/add.70119 (PMC12529237; doi:10.1111/add.70119)
Supplement: Supplementary file 1 — Figure S1. Study design and details on when measures were collected. Figure S2. Videos shown in the experimental condition. Table S1. Interactions between vaping/smoking status and intervention in predicting vaping harm perceptions, adjusting for covariates. [file ADD-120-2202-s001.docx]

**Supplementary Figure 1. Study design and details on when measures were collected.**

**
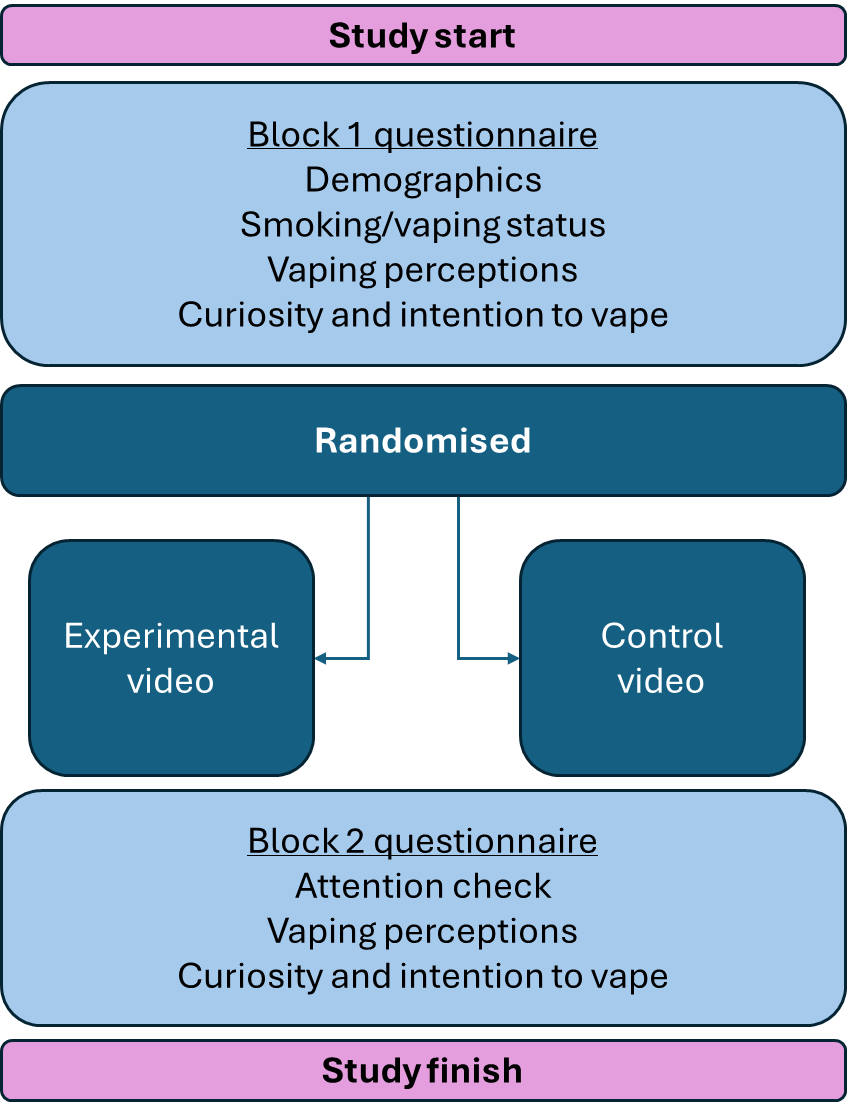
**

**Supplementary Figure 2. Videos shown in the experimental condition.**

| **Introduction to videos** | | | | | |
| --- | --- | --- | --- | --- | --- |
| 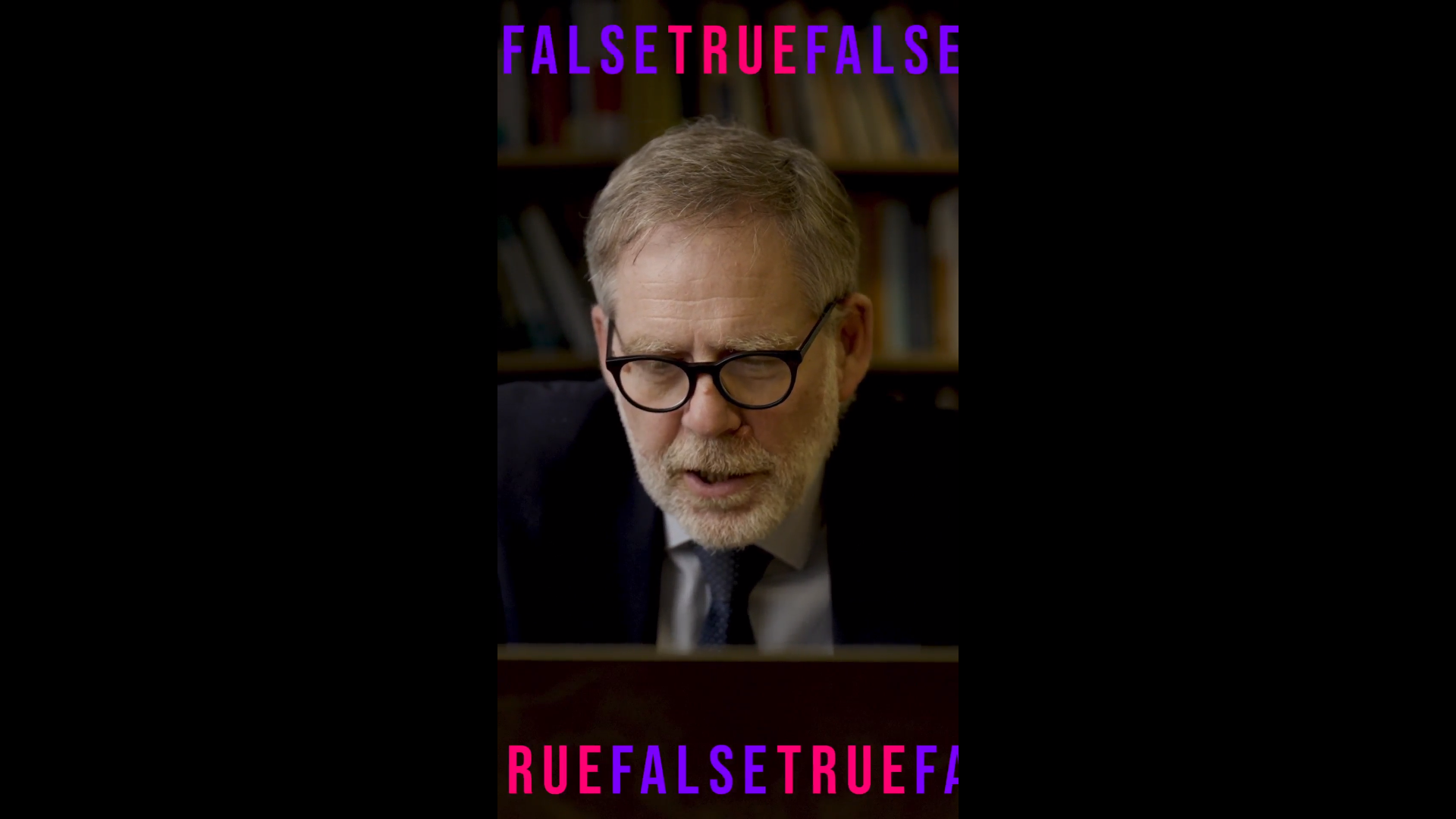 | | | | | |
| **Vaping is as harmful as smoking** | | | | | |
| 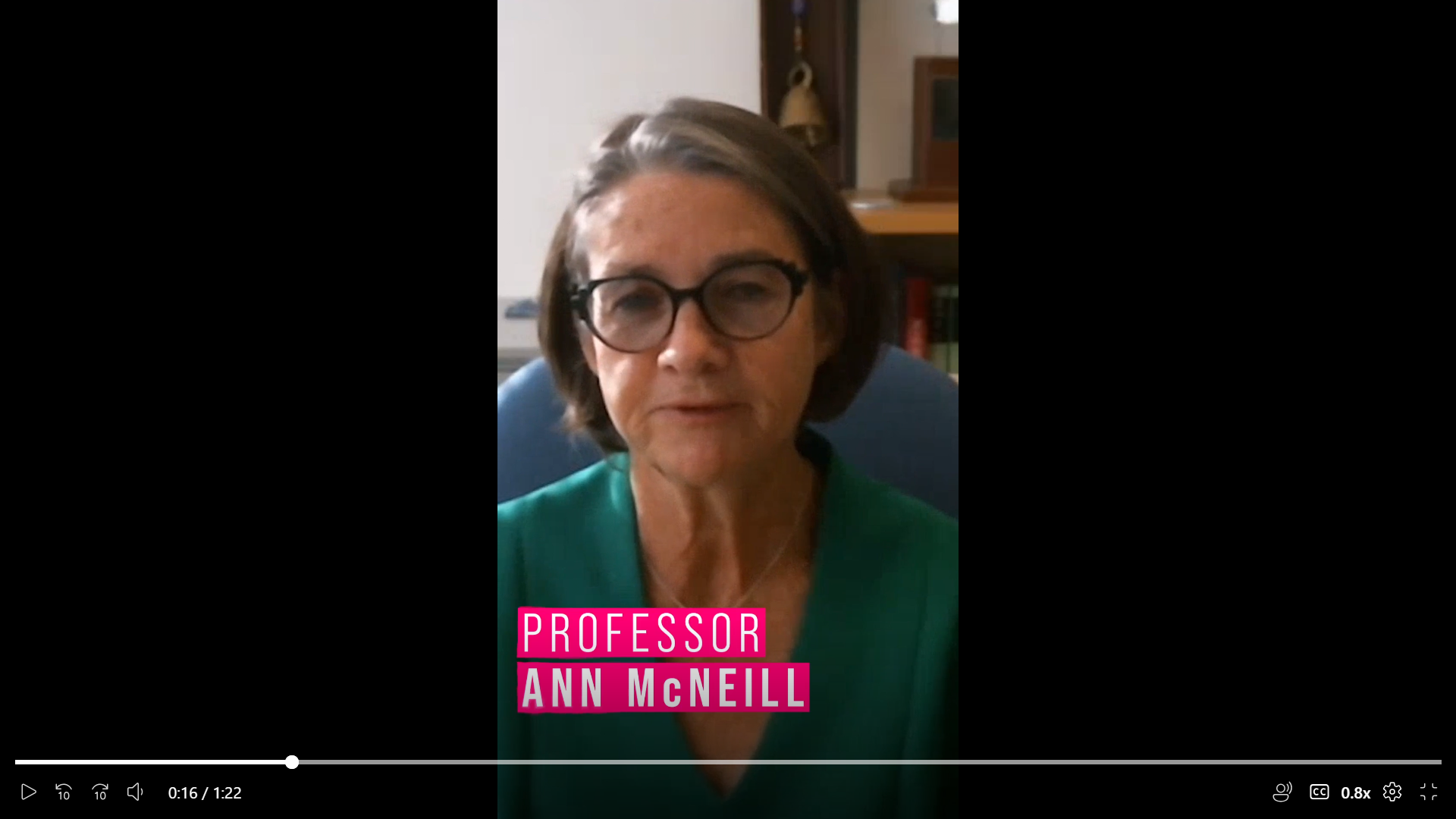 | | 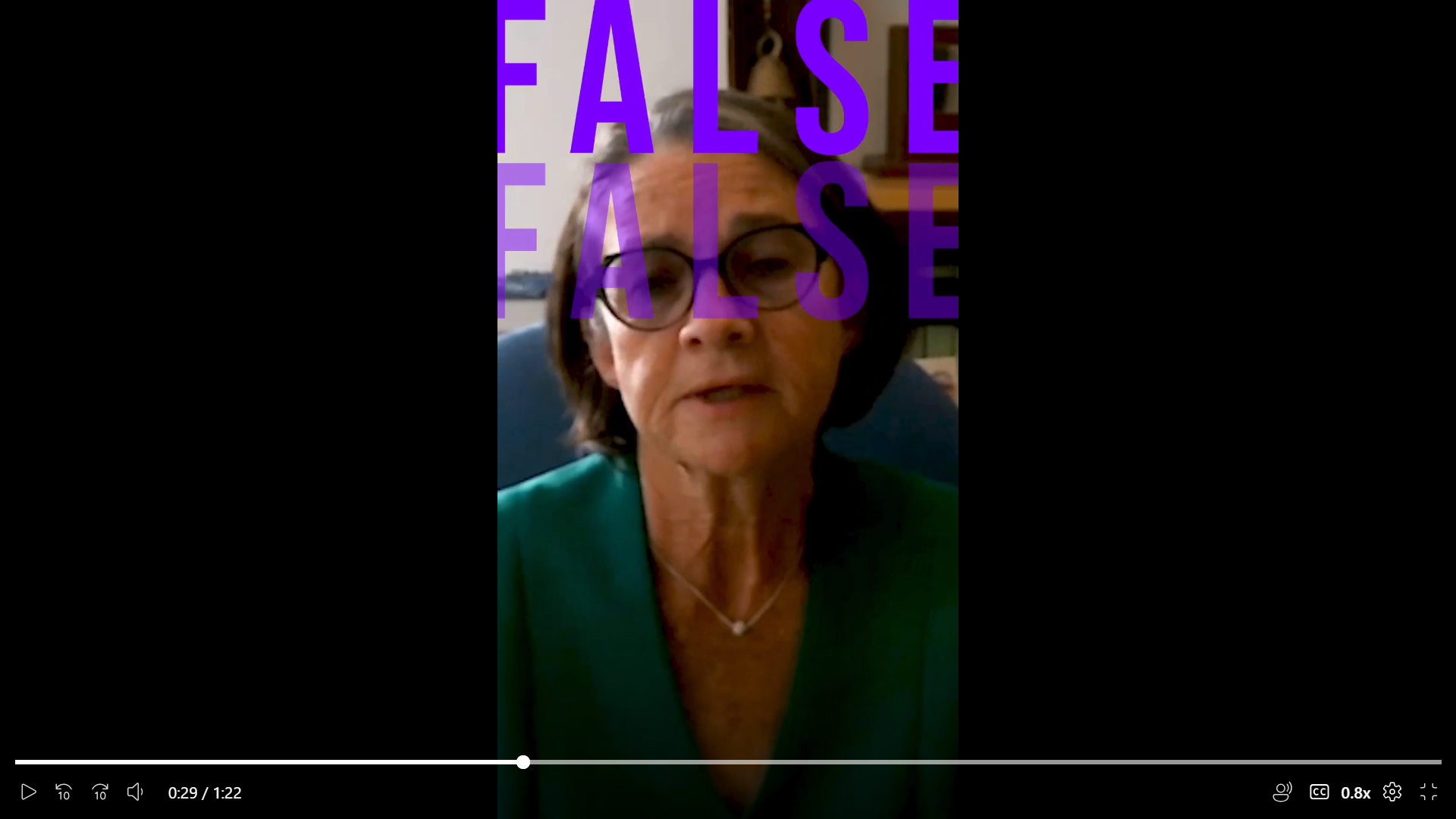 | | | 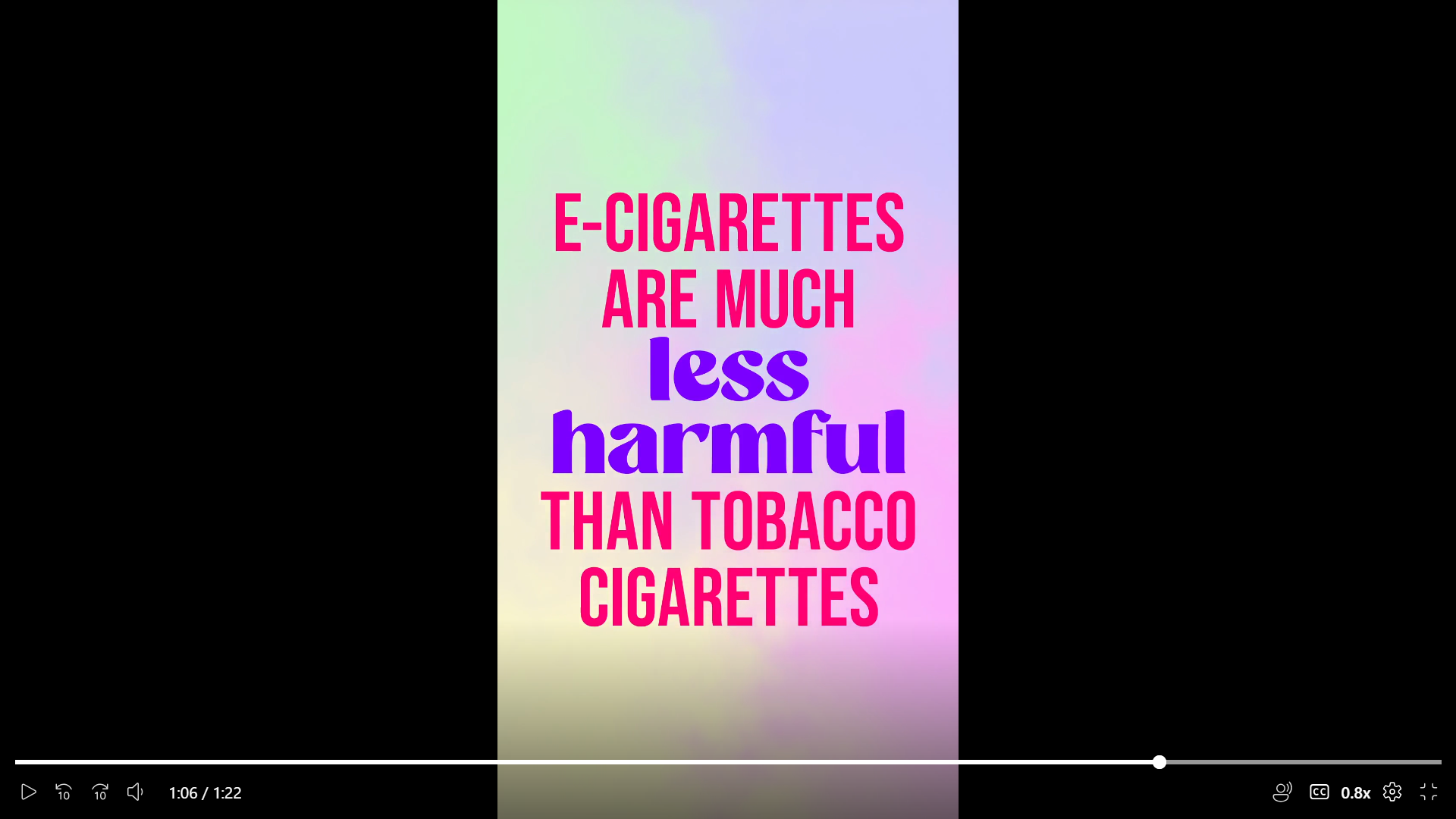 |
| **Vaping causes lung injury** | | | | | |
| 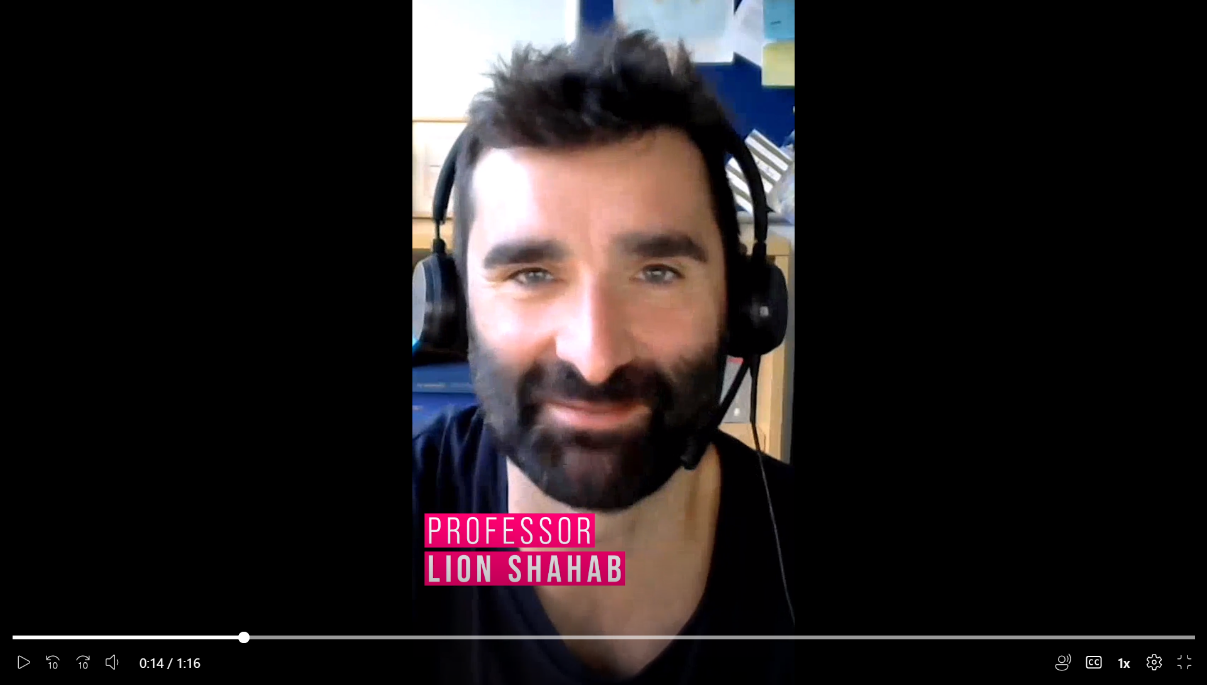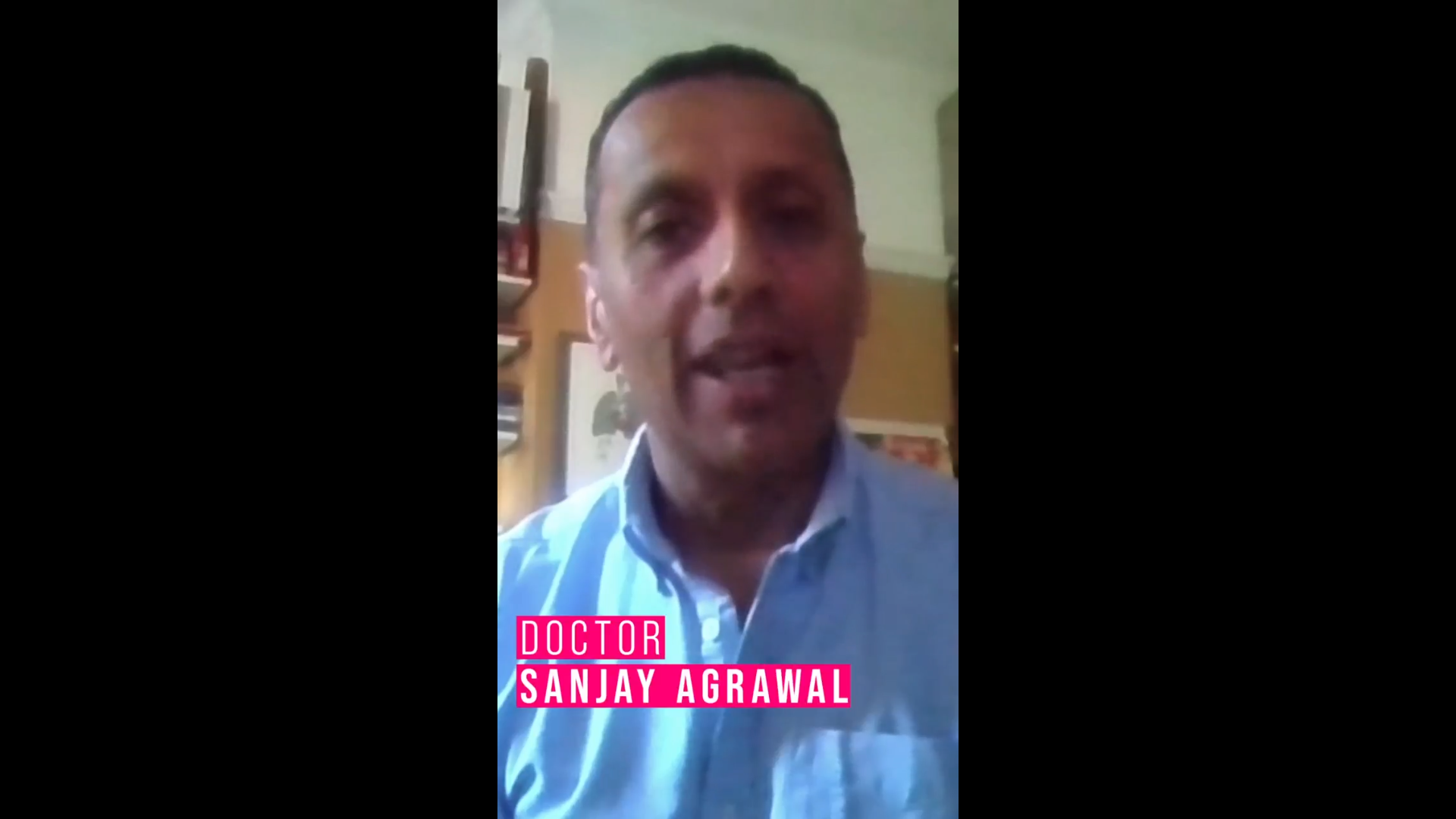 | | | 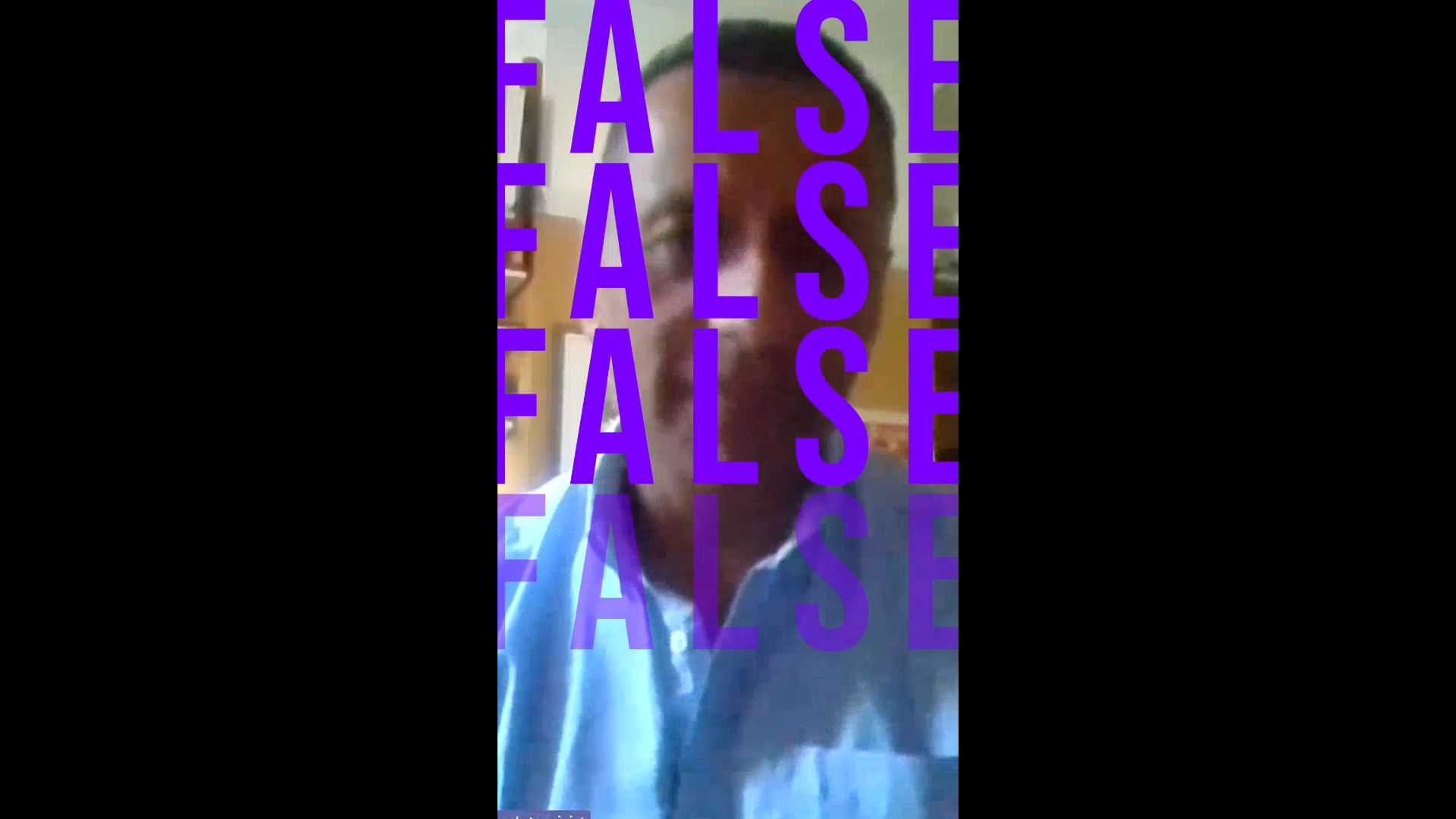 | | |
| **Vaping causes cancer** | | | | | |
| 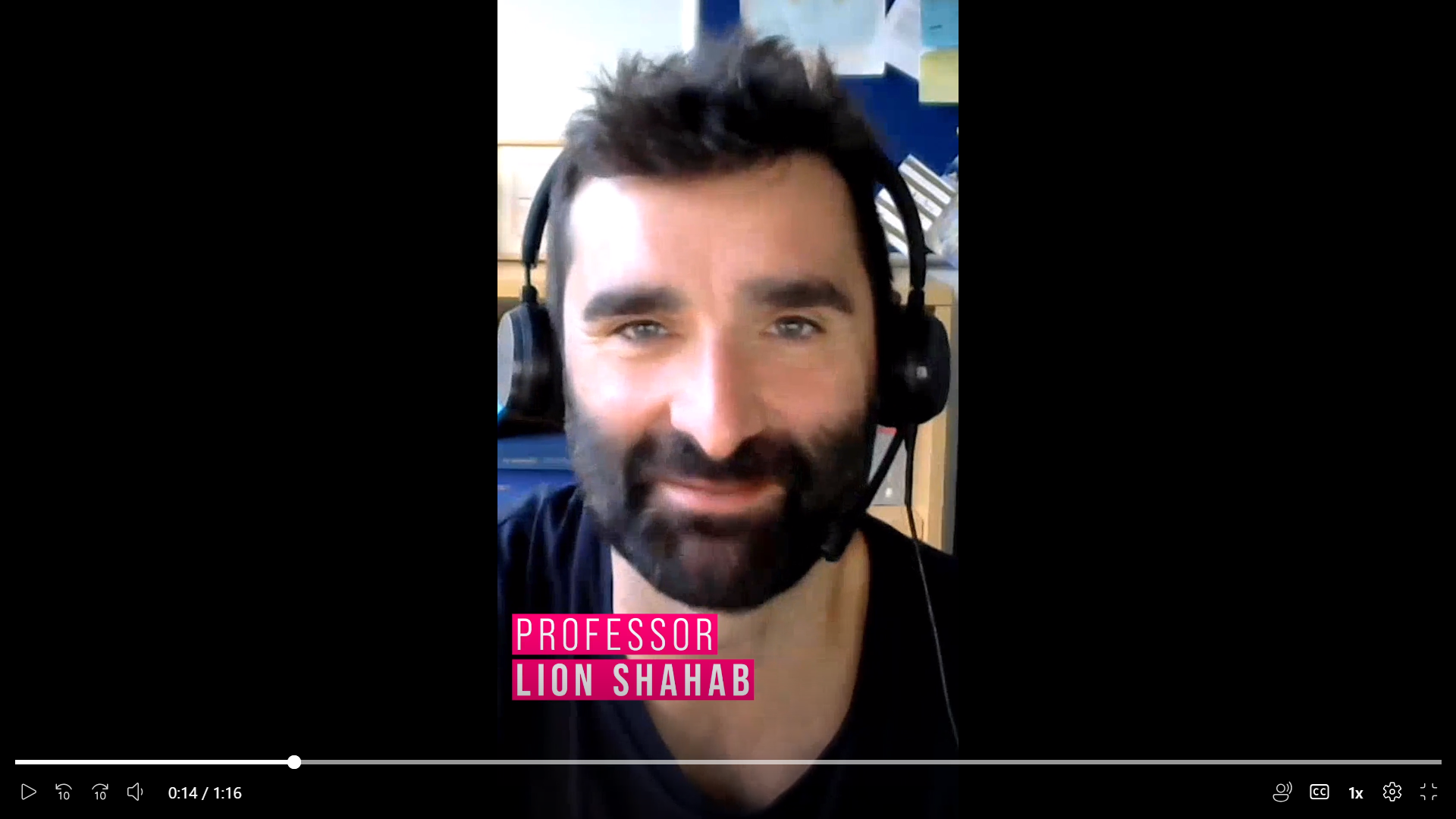 | | 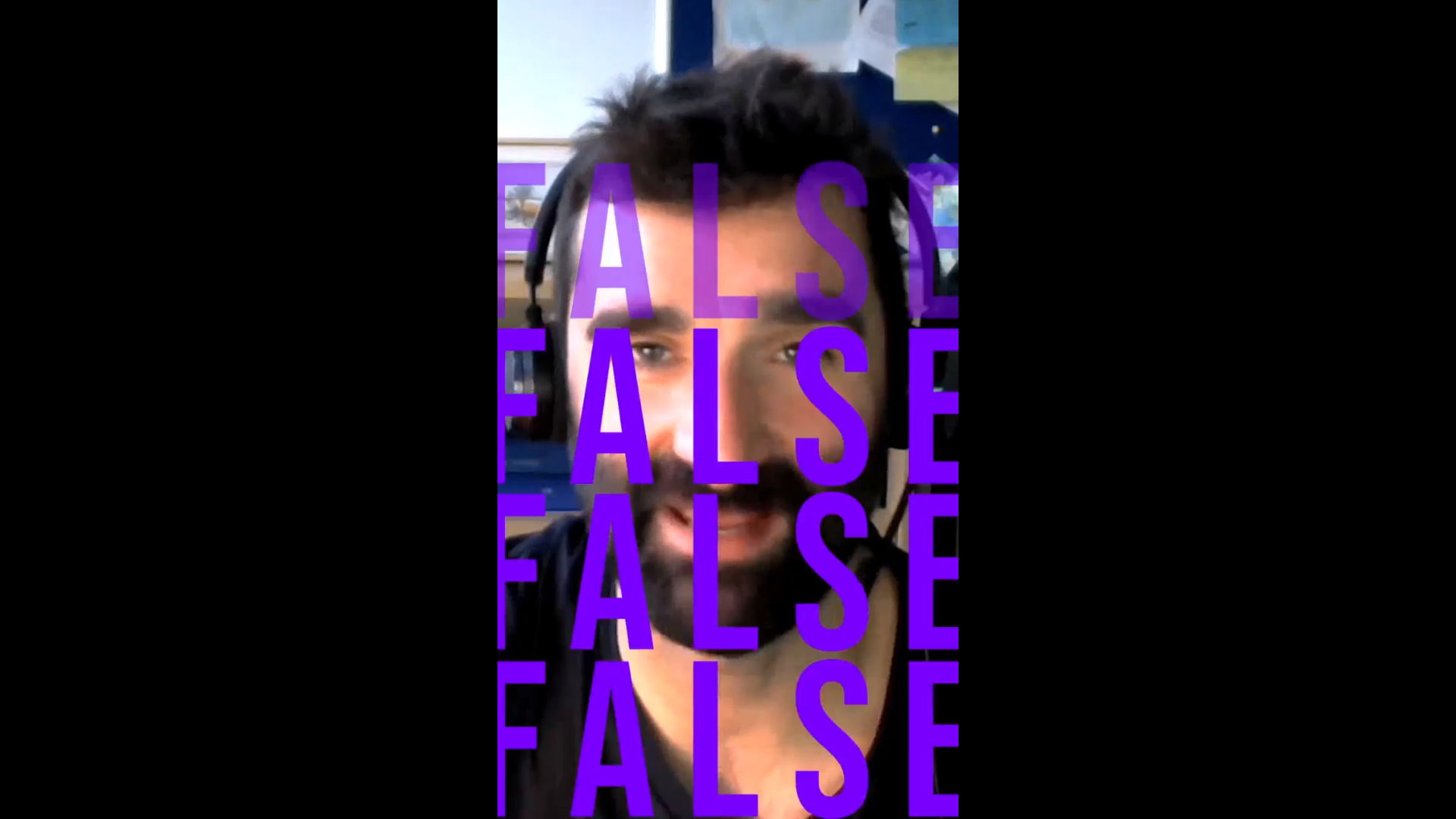 | | | 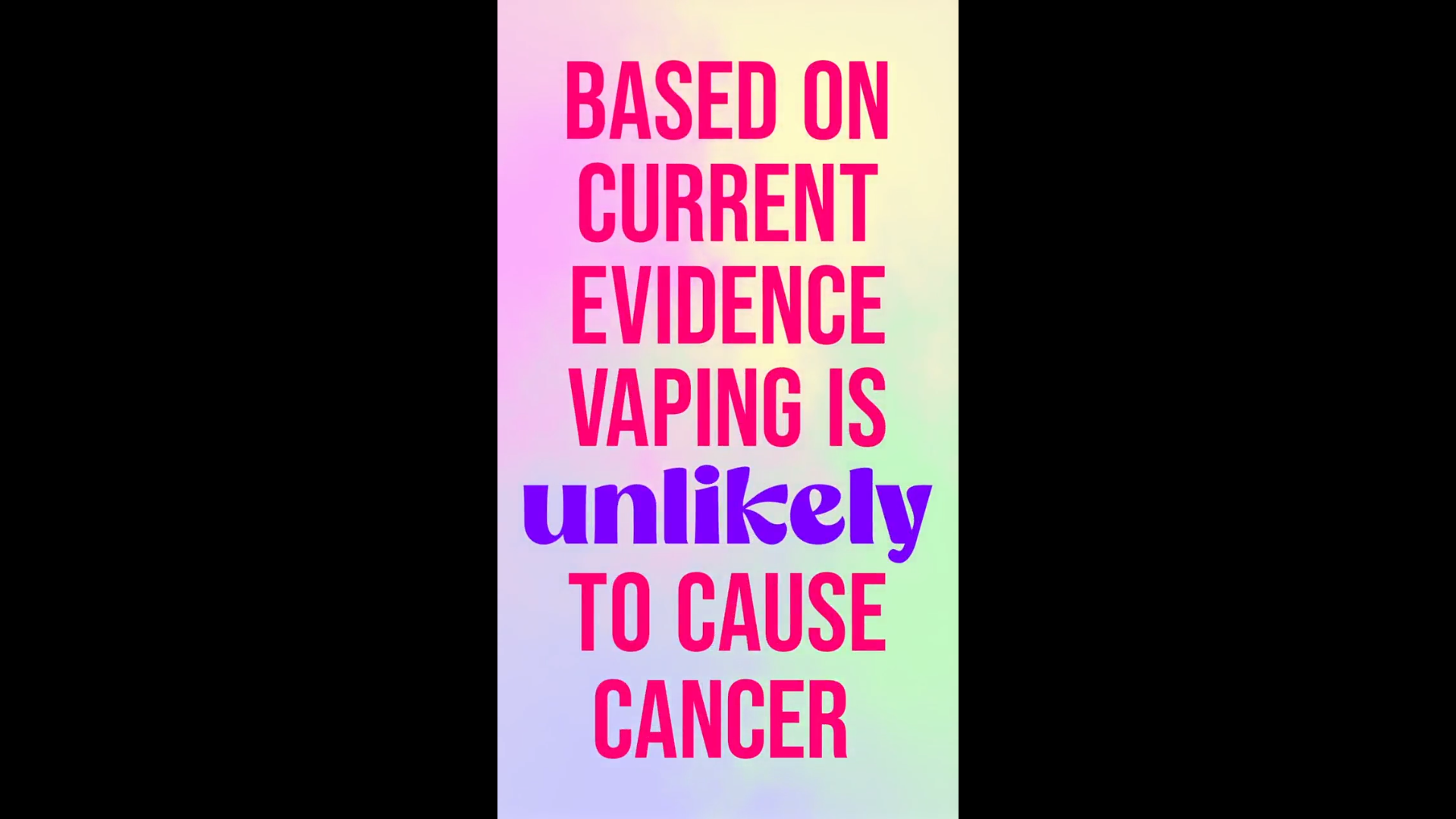 |
| **Nicotine is harmful** | | | | | |
| 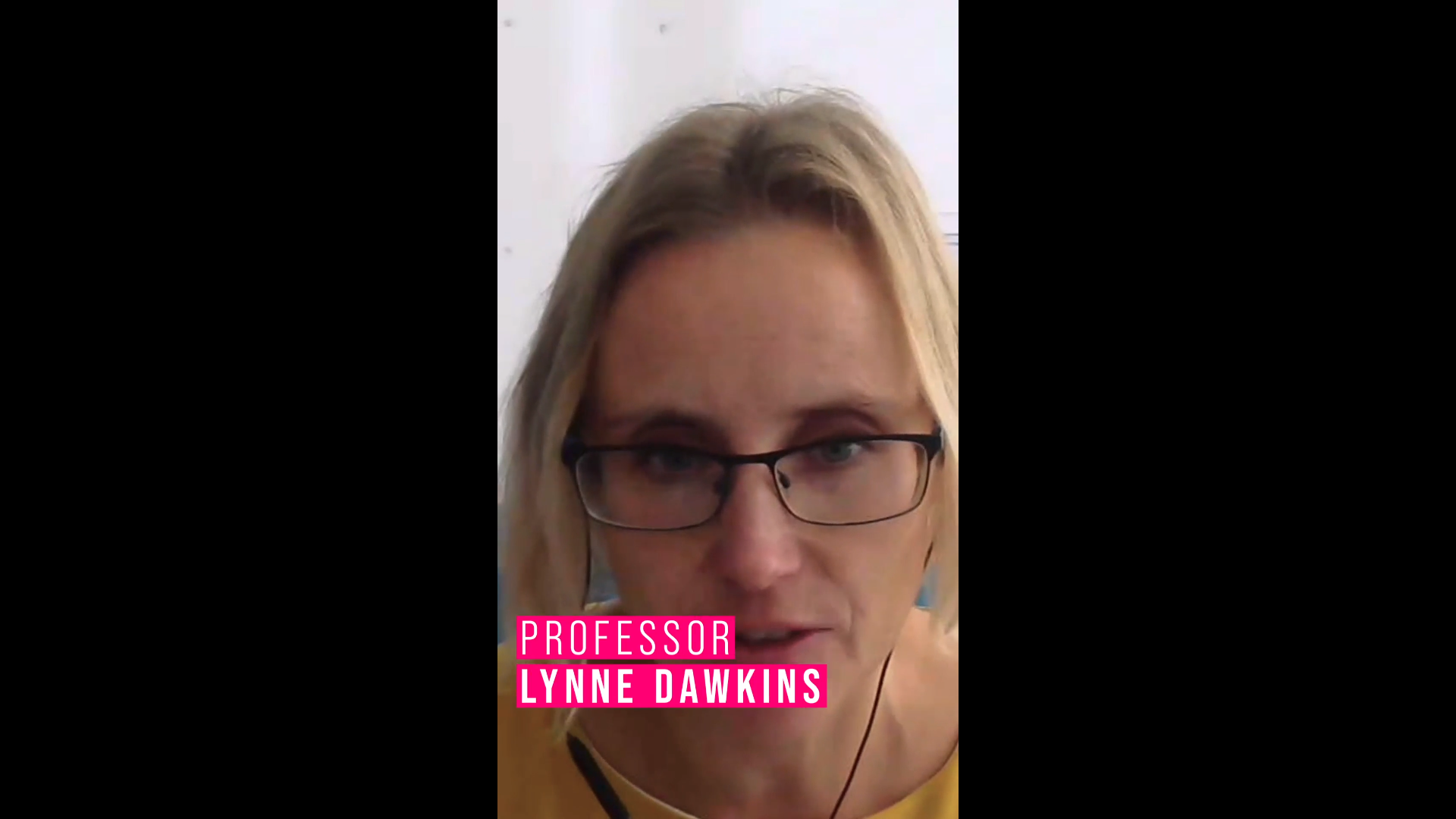 | | 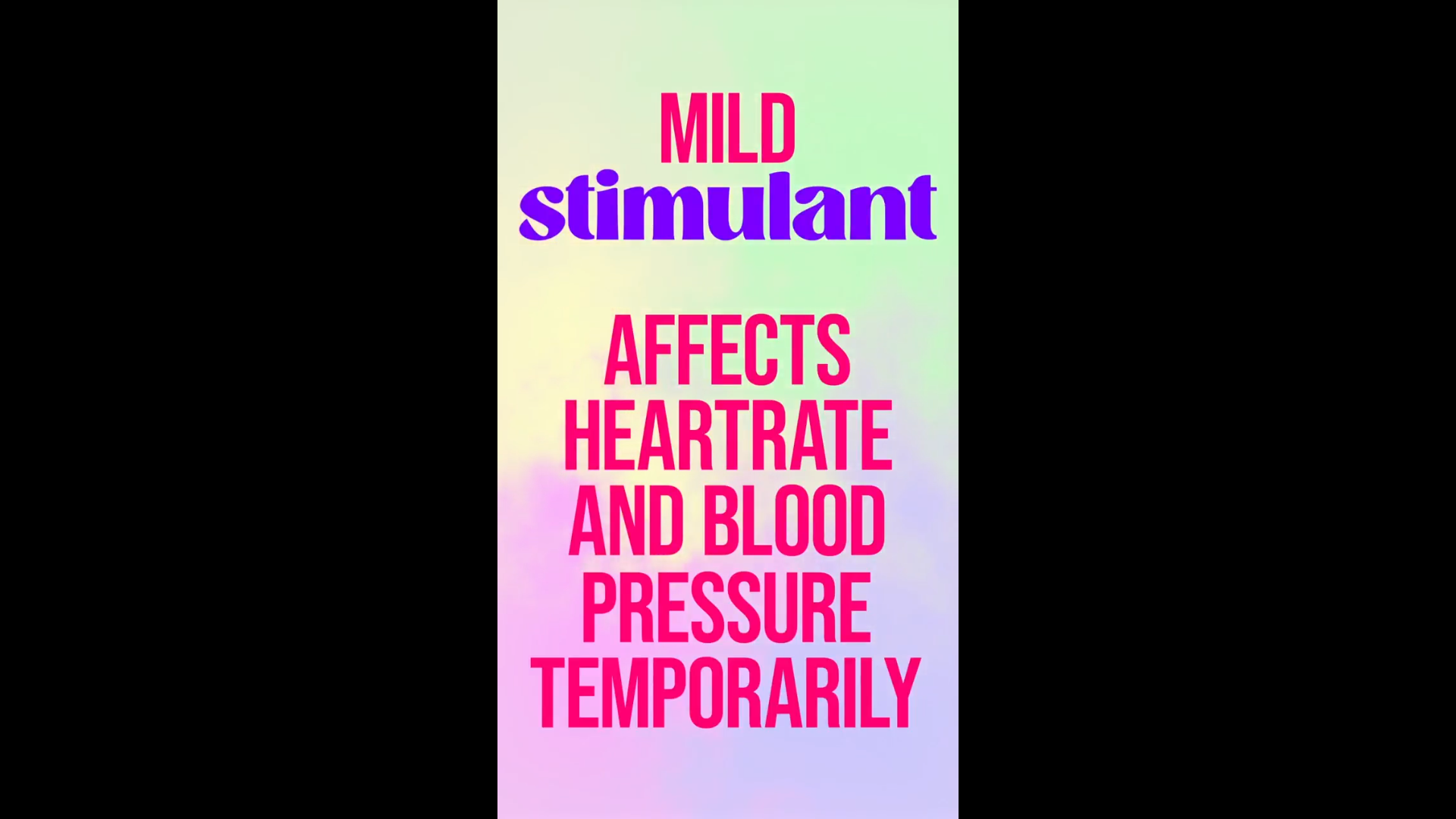 | | | 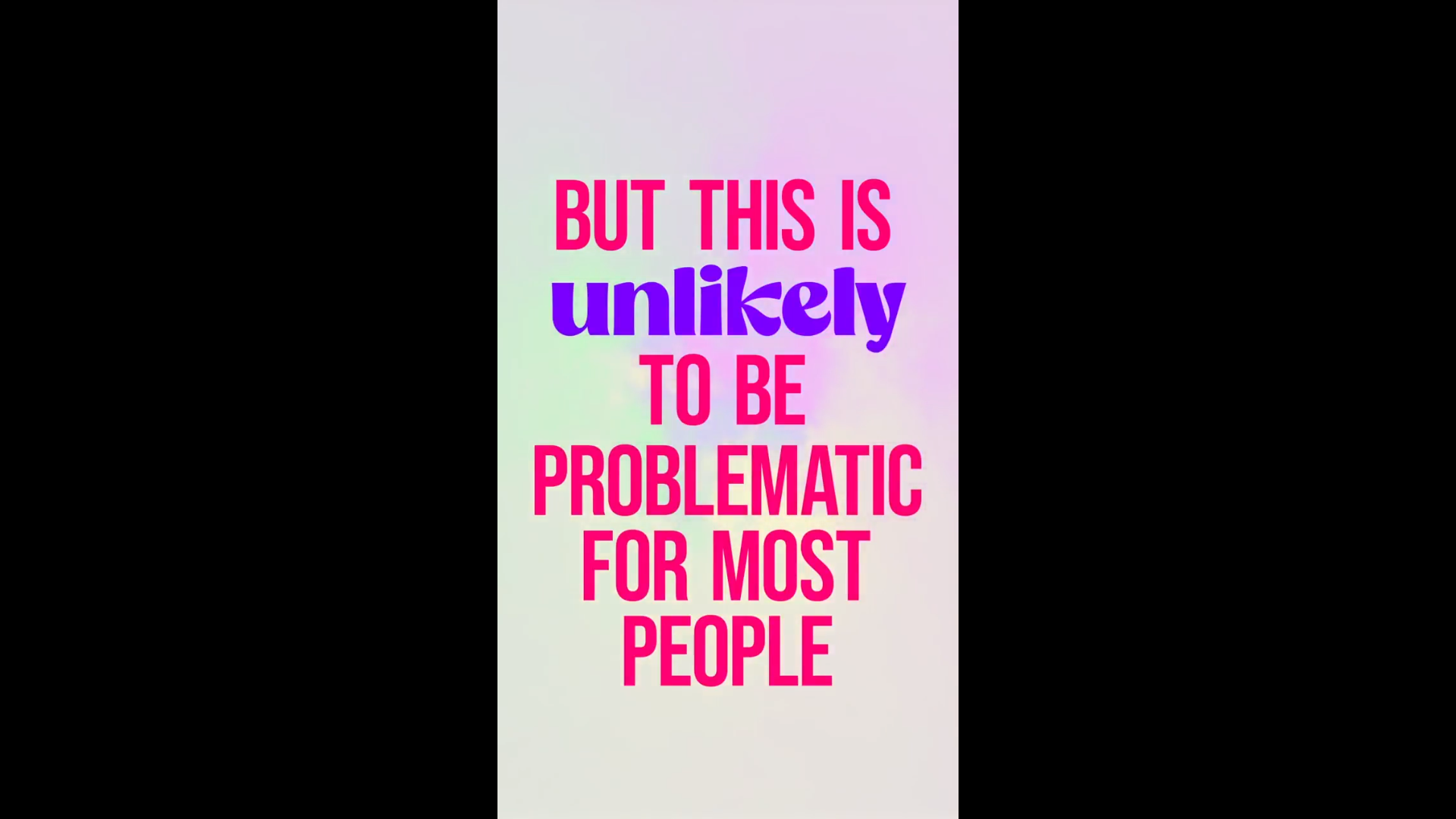 |
| **Pregnant women should not vape** | | | | | |
| 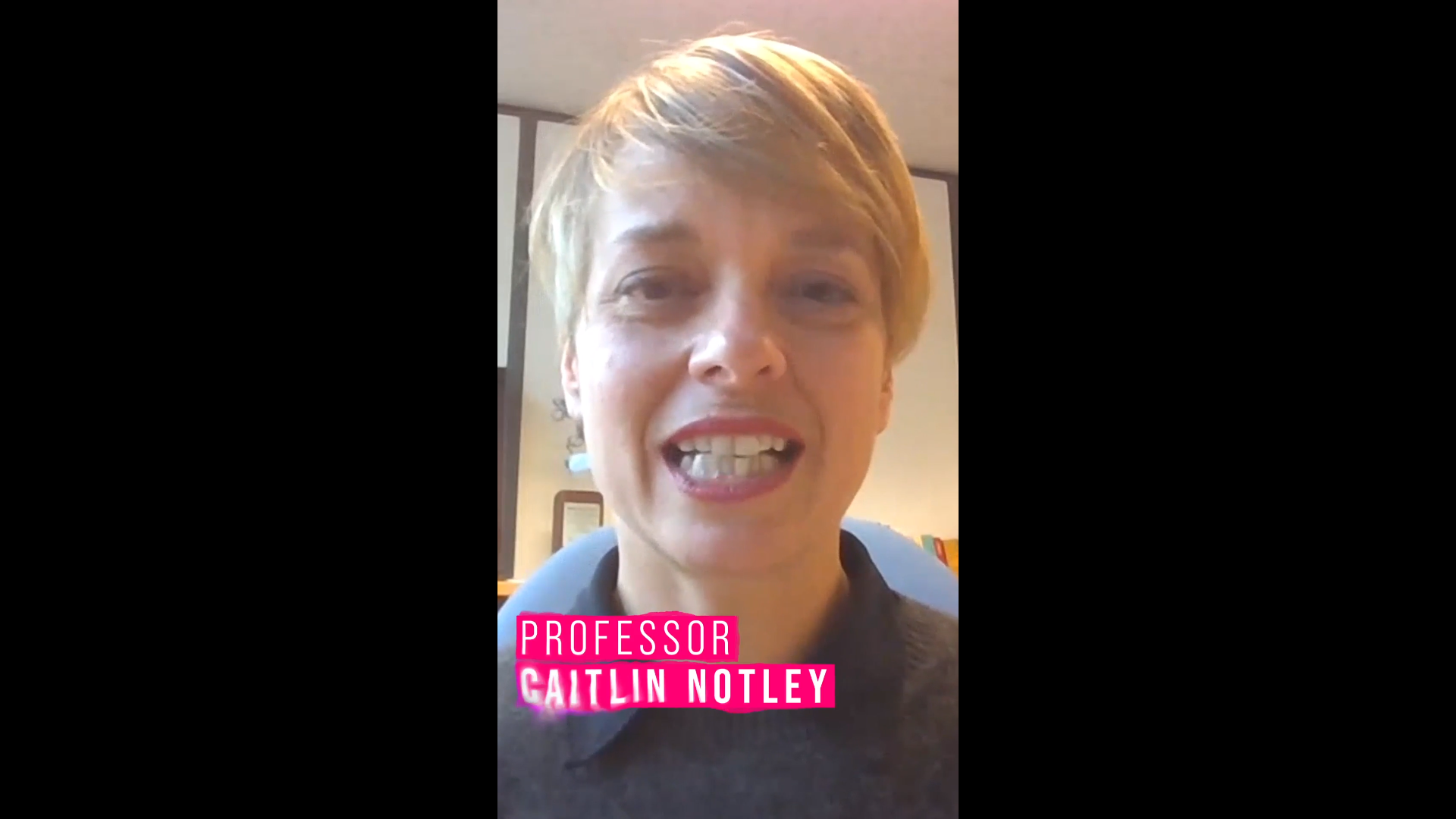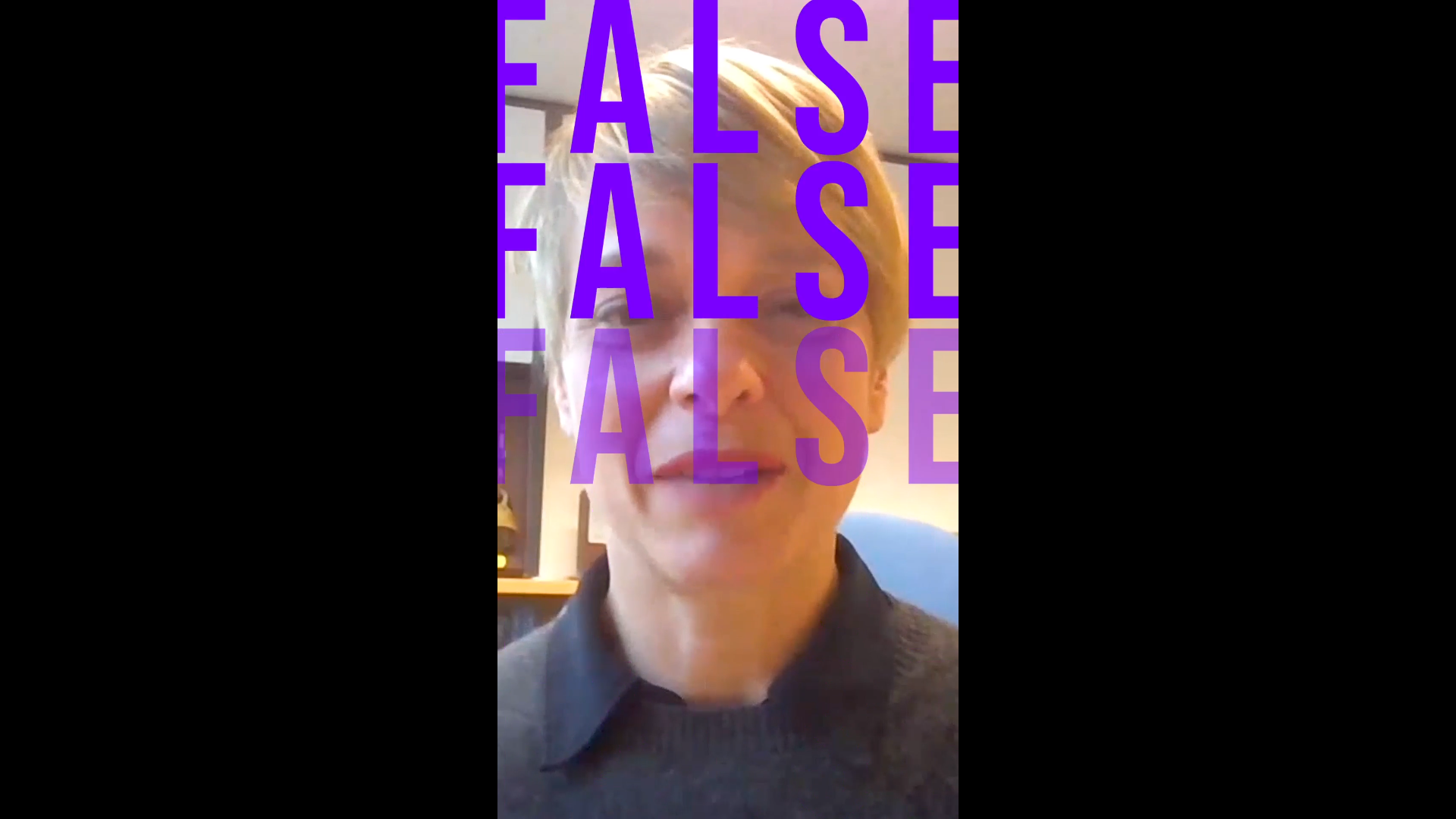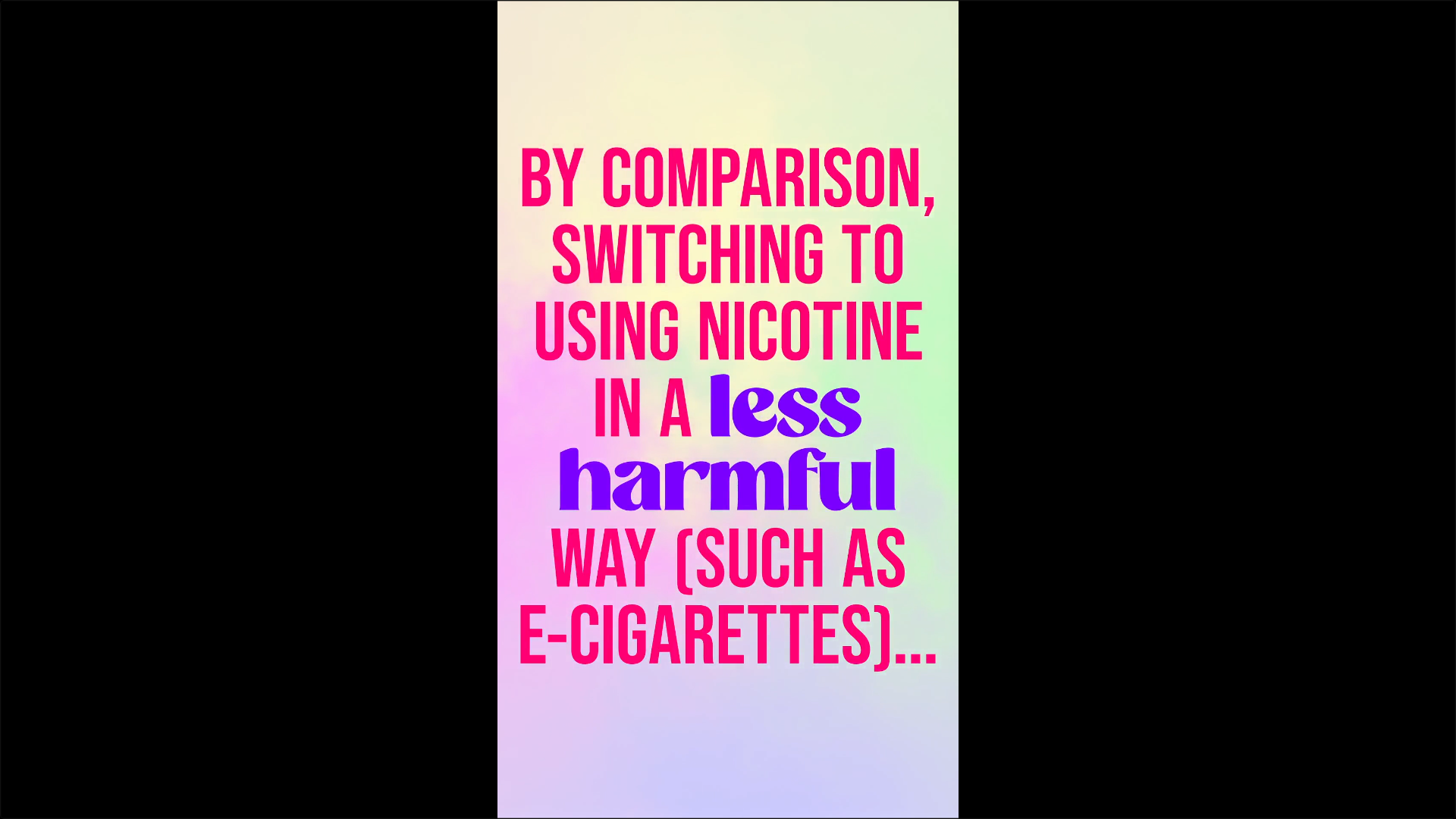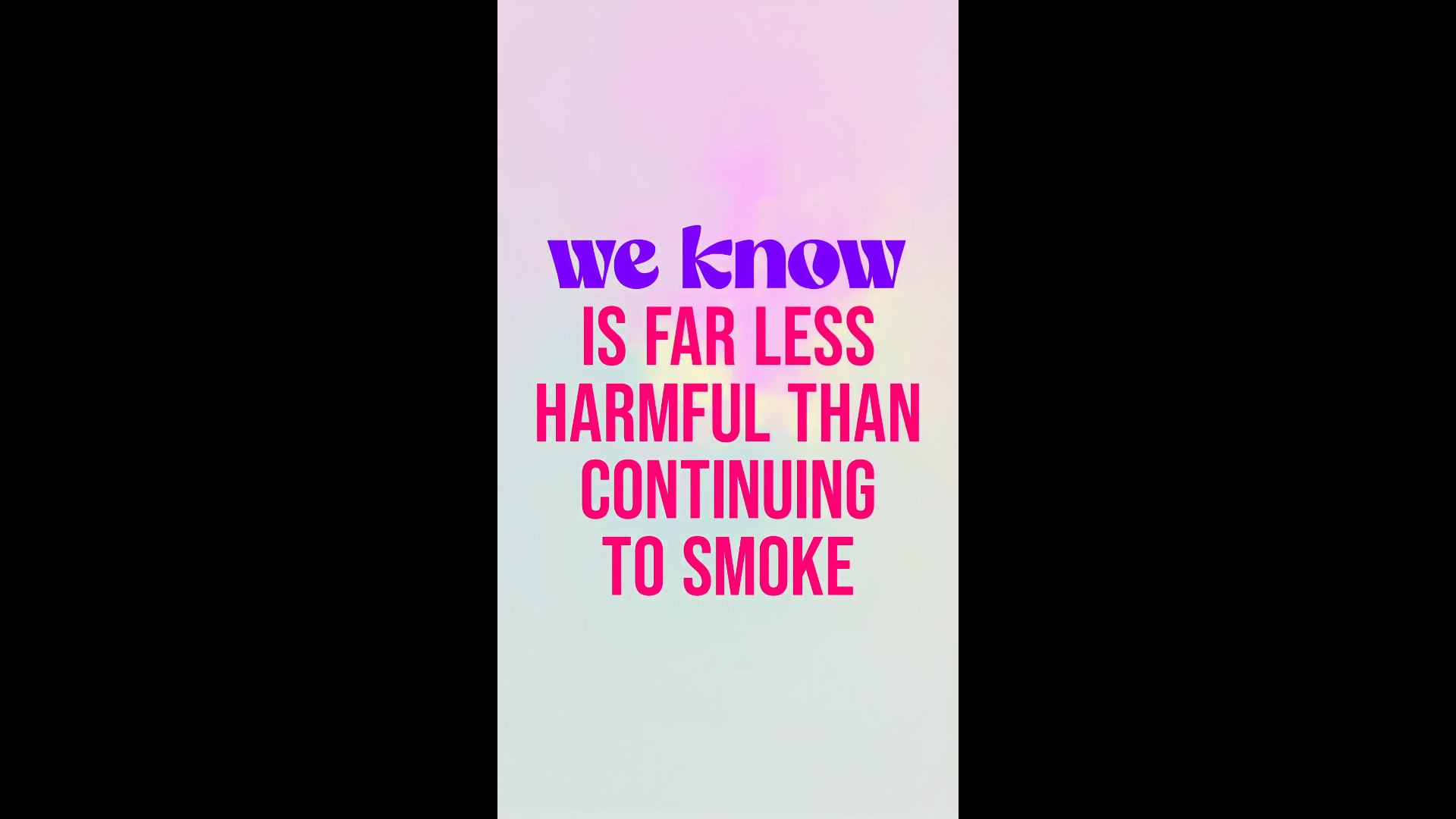 | | | | | |
| **Vaping is just swapping one addiction for another** | | | | | |
| 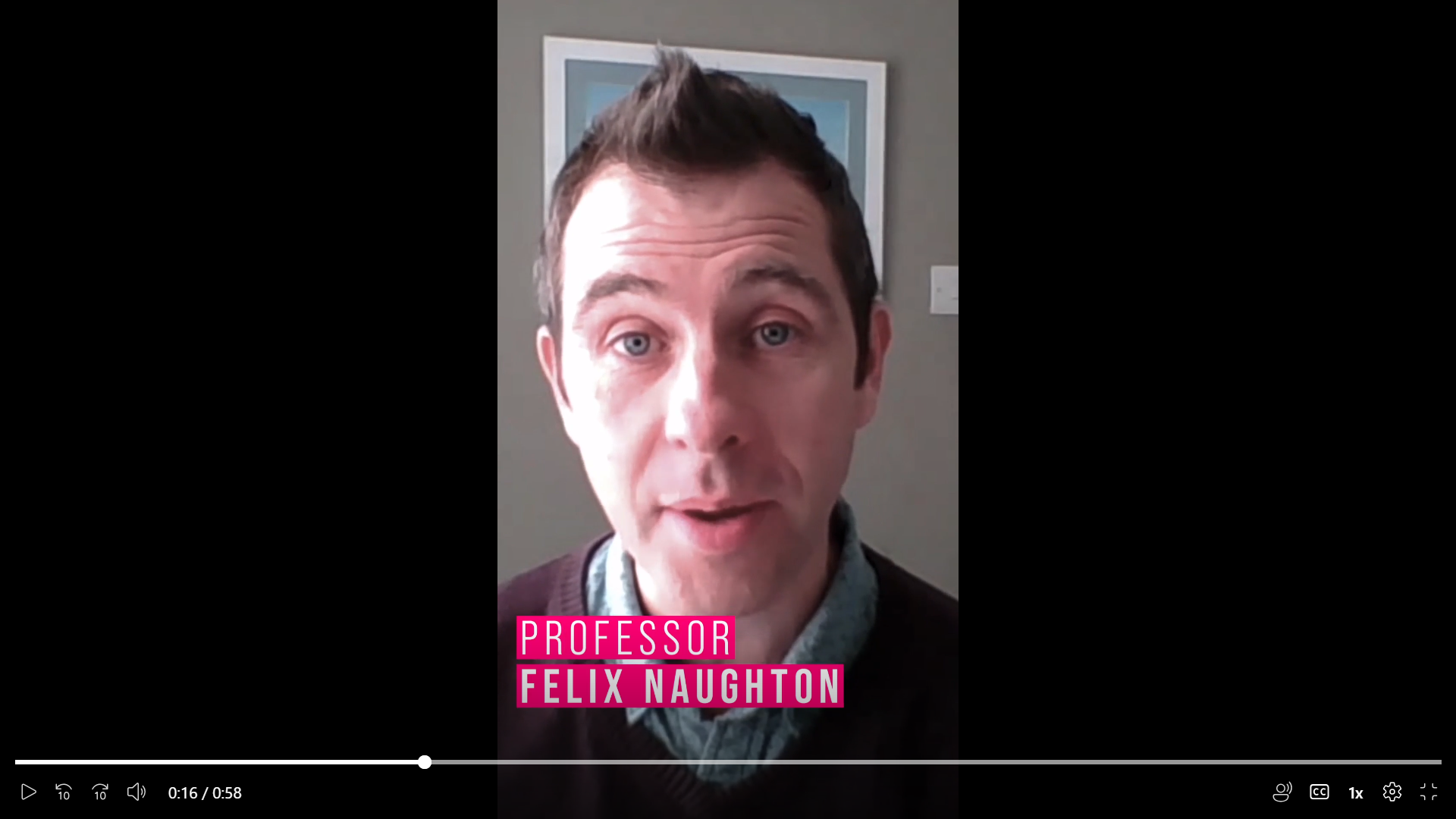 | 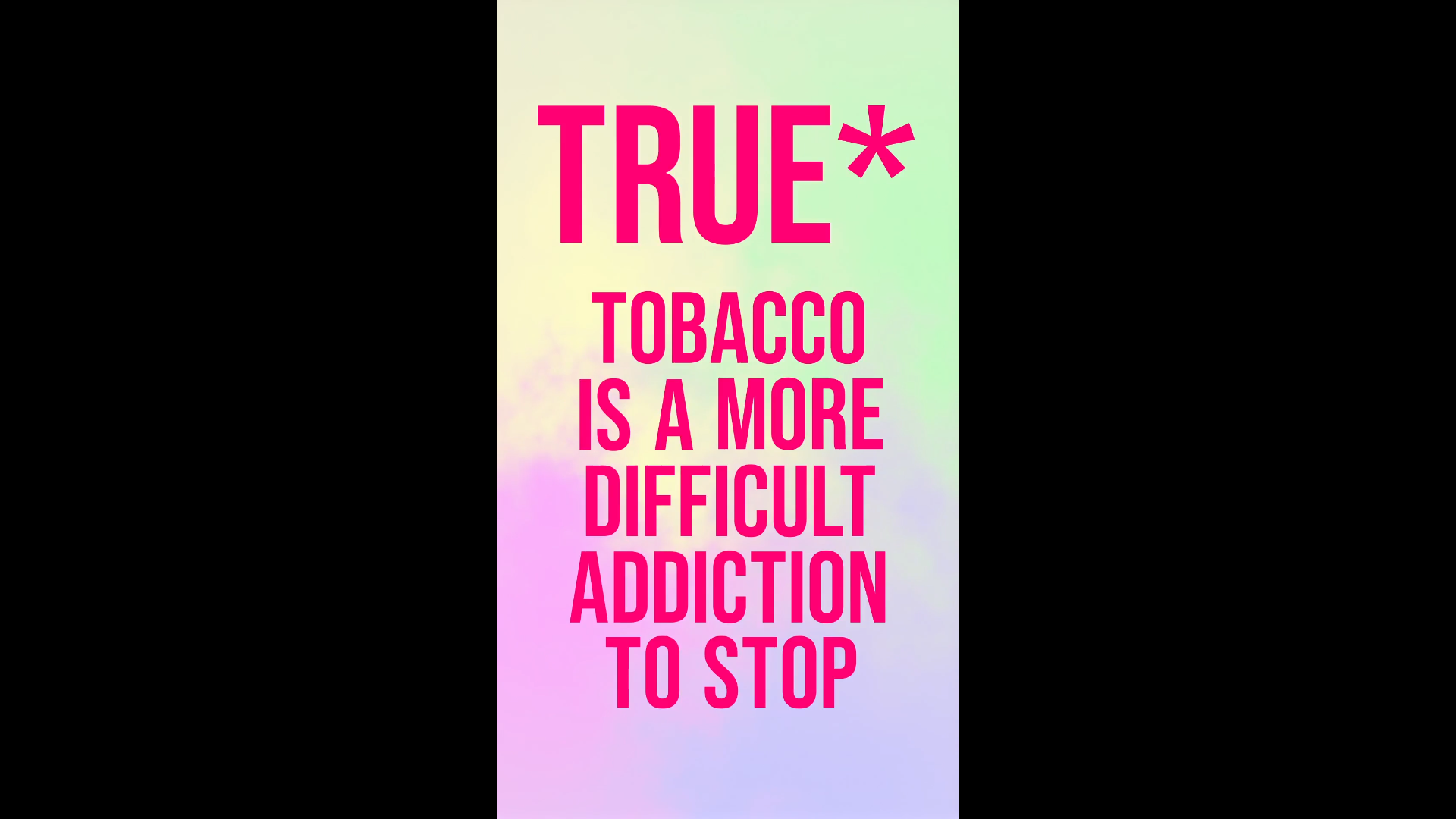 | | | **“*partially true…and it’s much more harmful than vaping. Vaping is much lower risk.”** | |
| **Nicotine vapes will not help you quit smoking** | | | | | |
| **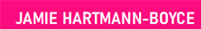**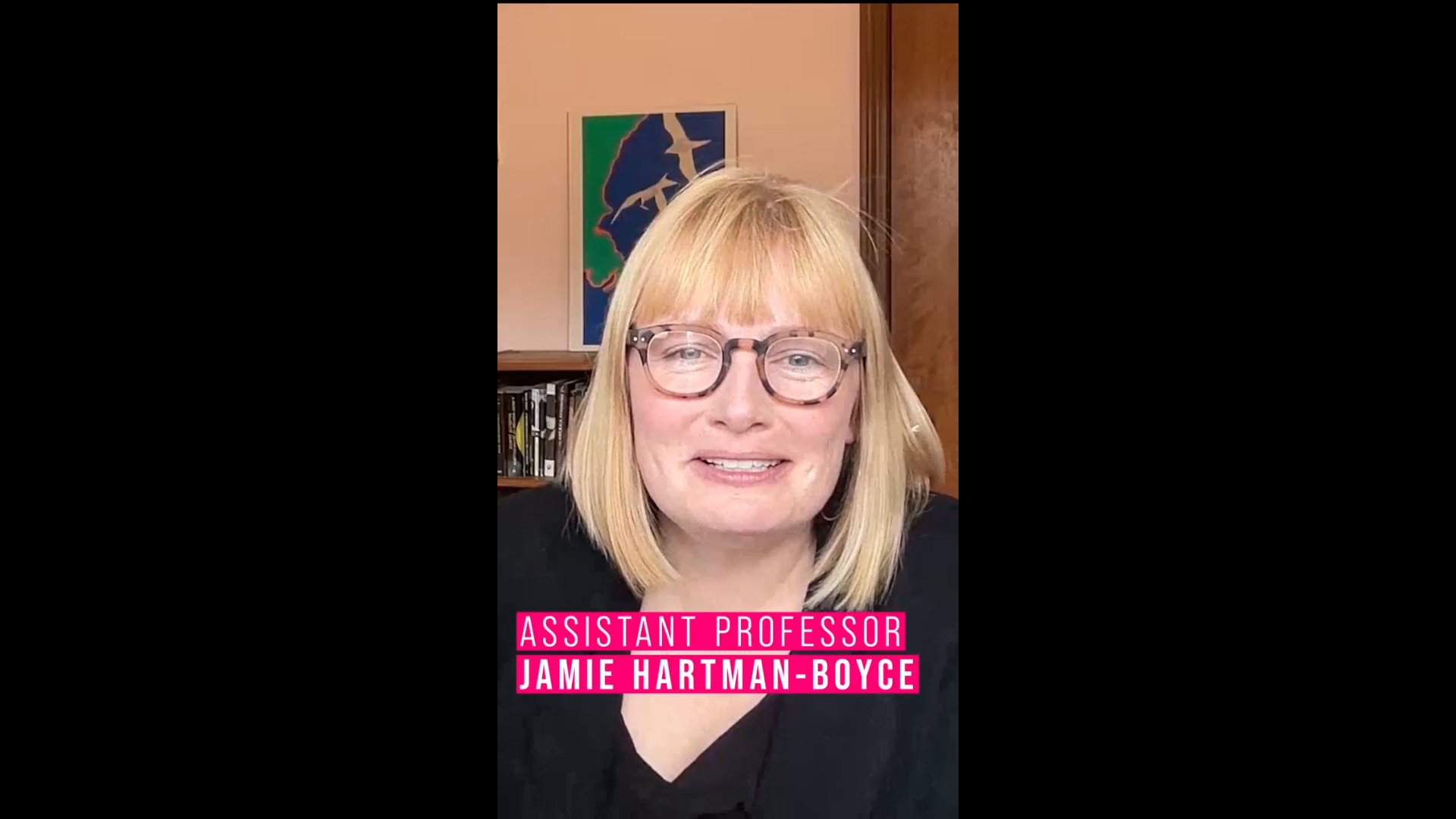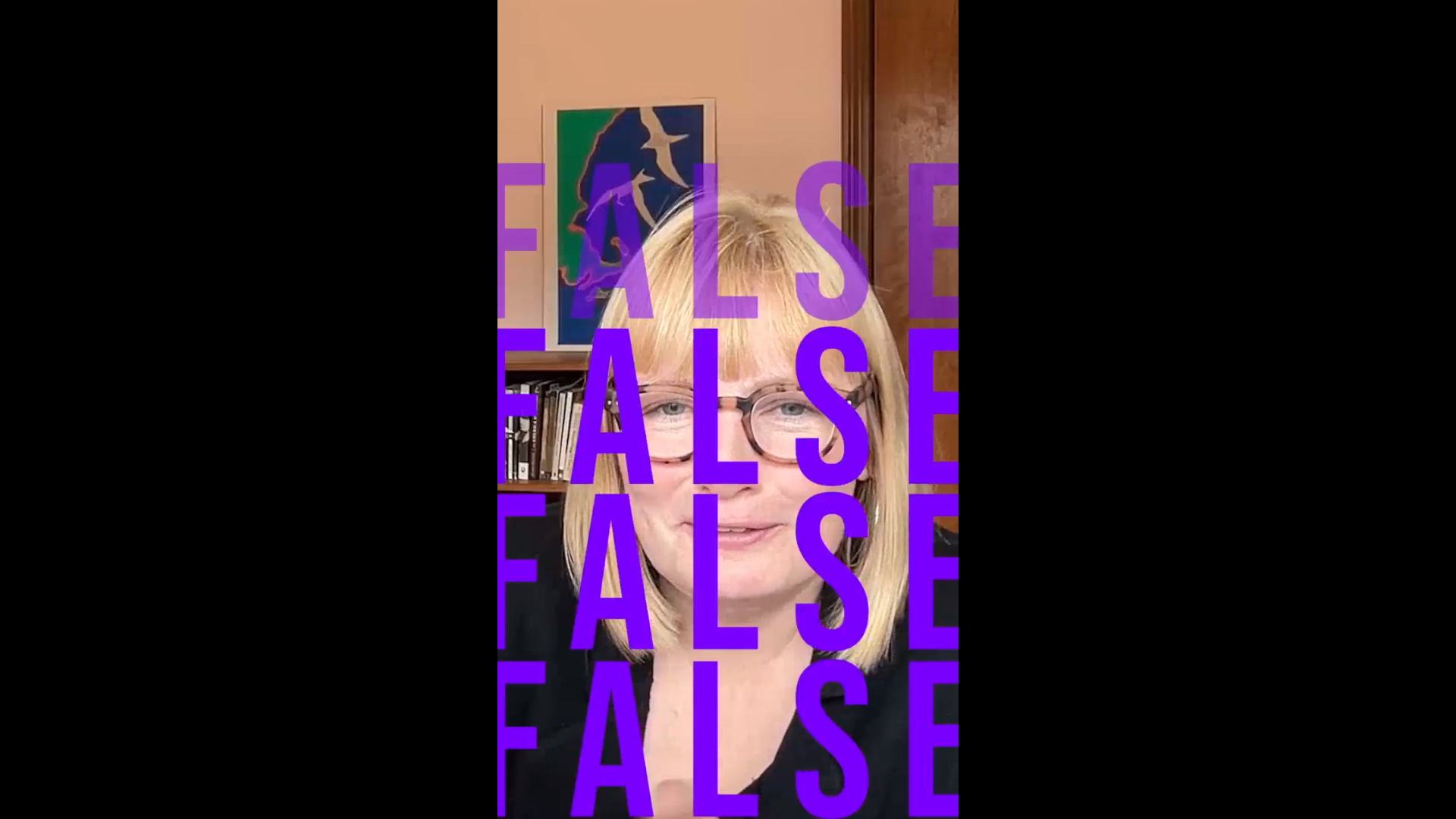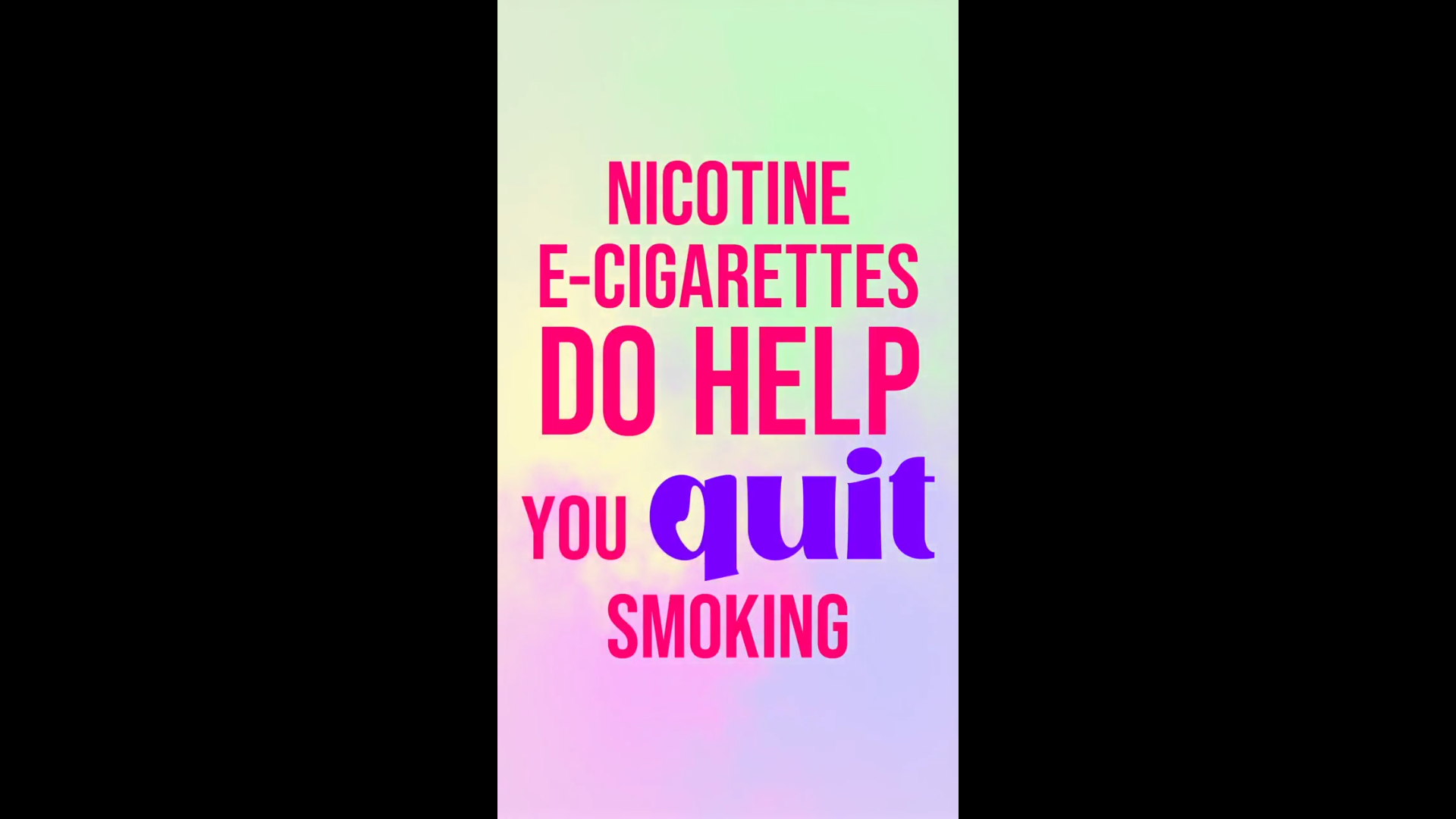 | | | | | |
| **Vaping has no place on the NHS** | | | | | |
| 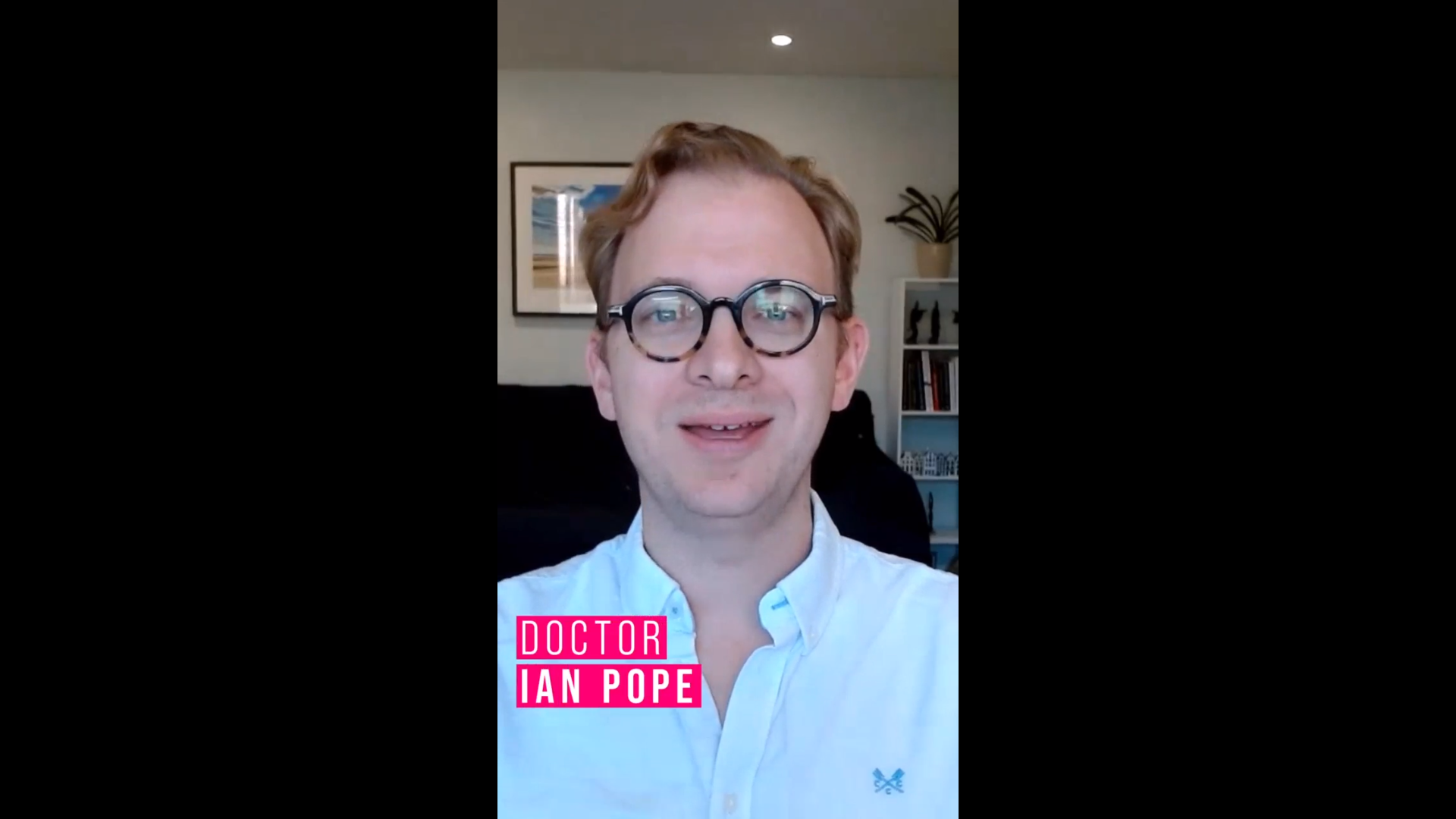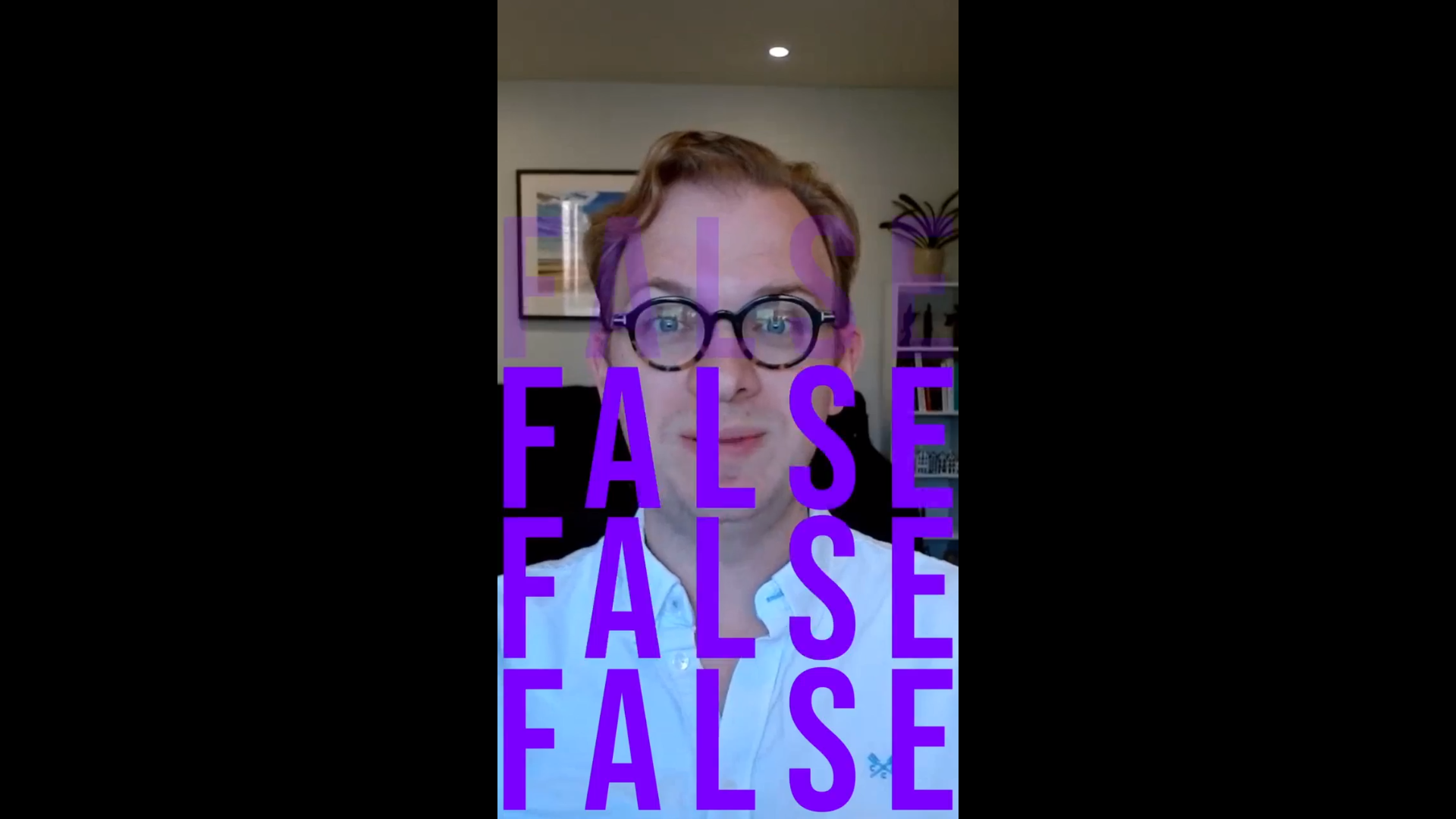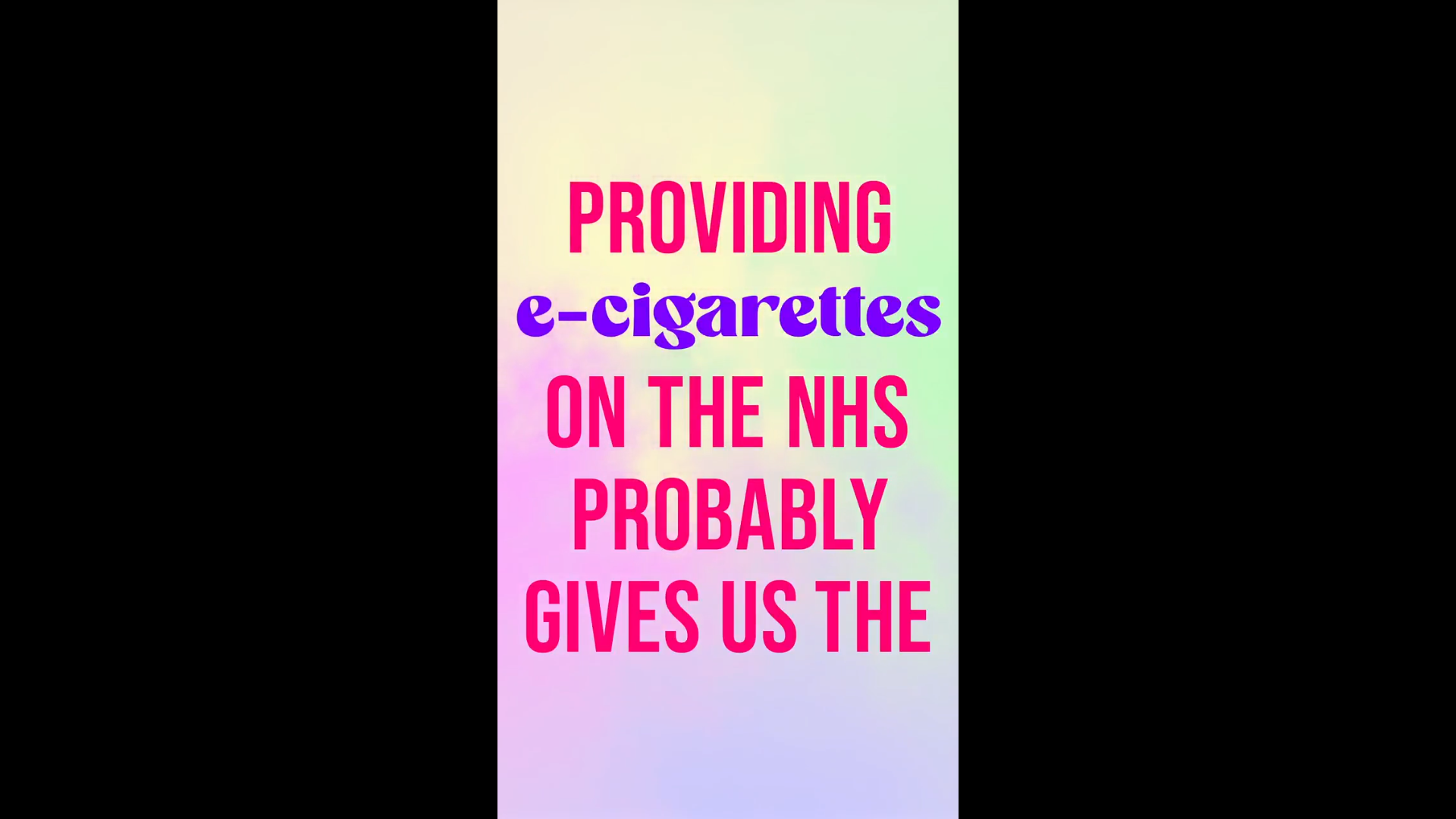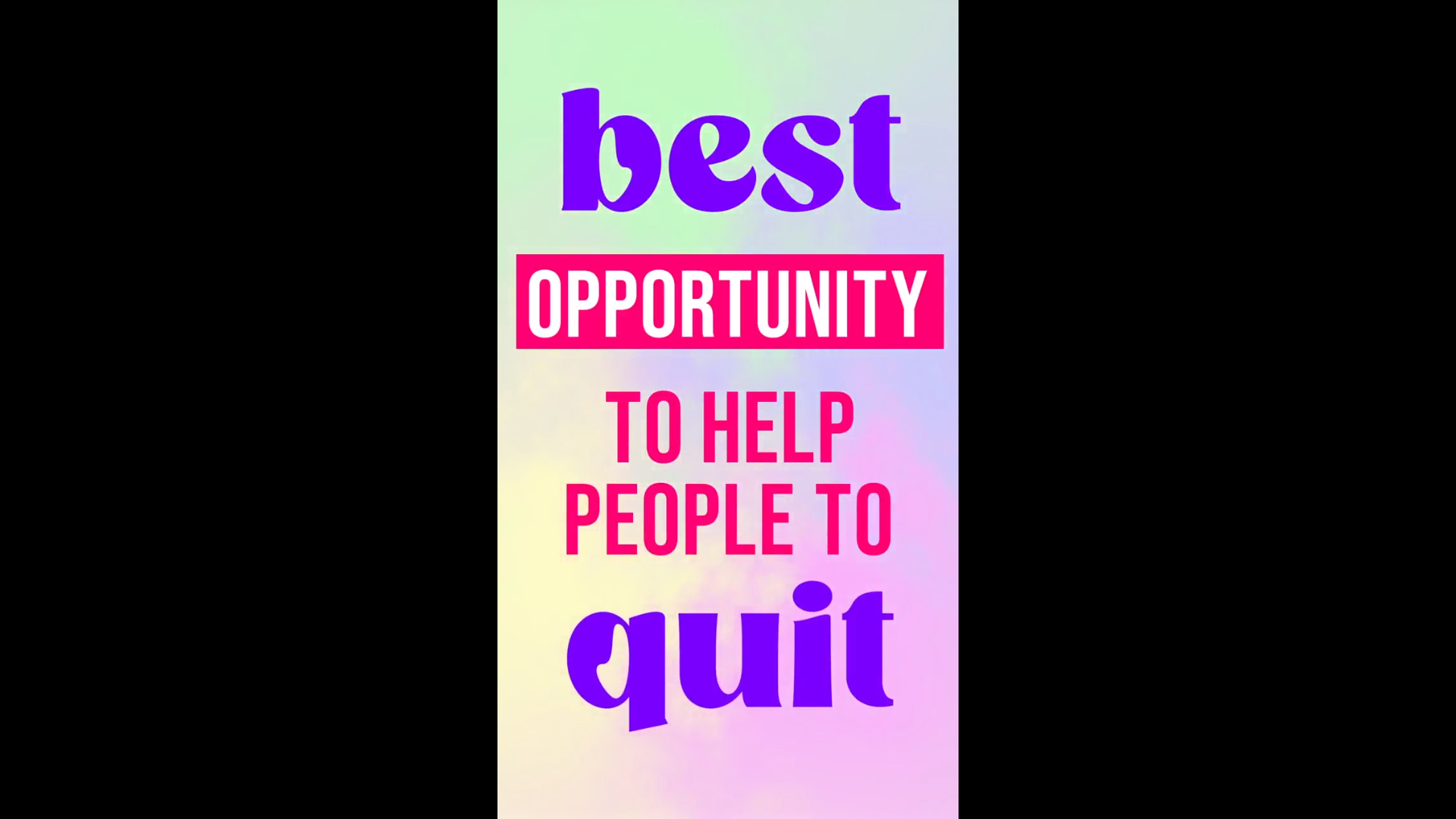 | | | | | |

**Transcripts**

**Vaping is as harmful as smoking**

00:00:08 MD

Is vaping as harmful as smoking?

00:00:13 AM

Hello, I'm Professor Ann McNeill. I'm a Professor of Tobacco Addiction at Kings College London.

00:00:20 MD

OK. Professor Ann McNeill. True or false? Vaping is as harmful as smoking.

00:00:28 AM

False, that’s certainly not the case at all. We've been looking at the evidence now for nearly ten years on e-cigarettes, both absolute risks and relative risks to cigarette smoking. When we started doing it, we were basically looking at what was in the liquids compared with cigarettes, and then what was in the e-cigarrete vapour or aerosol compared with cigarette smoke, and then in our most recent report last year, we were able to look in depth at biomarkers and collectively the evidence is very clear that e-cigarettes are much less harmful than tobacco cigarettes. That doesn't mean they're risk free, but a clear message should be given to smokers that e-cigarettes are substantially less harmful than tobacco cigarettes.

**Vaping causes lung injury**

00:00:08 MD

Does vaping cause lung injury?

00:00:14 SA

So I am a respiratory and intensive care doctor and I've been a consultant now for over 20 years.

00:00:22 MD

OK, Sanjay, vaping causes lung injury, true or false?

00:00:28 SA

False. The vast majority of harm to the lungs from nicotine containing products actually comes from tobacco. So we know, therefore, that vaping is much safer than tobacco smoking. There are 4.7 million people who use vapes in the United Kingdom, we estimate. And, you know, I work on intensive care unit and I'm yet to see a vaping related lung injury from those 4.7 million people.

**Vaping causes cancer**

00:00:08 MD

Does vaping cause cancer?

00:00:13 LS

Hi. My name is Lion Shahab. I'm Professor of Health Psychology at University College London, and I have around 20 years of experience of working in the field of tobacco research, including most recently working on evaluating the effects of e-cigarettes, both on health outcomes, so exposure to harmful substances, but also as a tool to help people stop smoking.

00:00:37 MD

Professor Lion Shahab. Vaping causes cancer. True or false?

00:00:44 LS

False, based on the best available evidence to date, vaping is unlikely to cause cancer.

00:00:51 MD

I like that. So as a wise man once said, if the choice is between vaping and smoking, choose vaping. But if the choice is between vaping and fresh air, choose fresh air.

00:01:05 LS

Absolutely right, yes. Always choose fresh air. If you're not able to choose fresh air, then please vape rather than smoke.

**Nicotine is harmful**

00:00:08 MD

Is nicotine bad for your health?

00:00:13 LD

I'm Lynne Dawkins. I am a professor of Nicotine and Tobacco Studies at London South Bank University and I've been working in the field of nicotine and tobacco research for over 25 years.

00:00:26 MD

My question for you, I think I know the answer. Is nicotine harmful? I think the answer is mostly no. Is that right?

00:00:36 LD

OK, I'd say not particularly, especially when used in ways that doesn't involve smoking tobacco products and other products. It is a mild stimulant. So, it has an effect on heart rate and blood pressure, temporarily increasing those. But this is unlikely to be problematic for most people. People smoke for the nicotine, but they die from the carbon monoxide and the tar. So that's why nicotine replacement therapies literally replace the nicotine and are known to be effective and very safe.

00:01:10 MD

Professor Lynne Dawkins, thank you very much.

**Pregnant women should not vape**

00:00:08 MD

Should pregnant women vape?

00:00:13 CN

My name is Caitlin Notley. I'm a Professor of Addiction Sciences at the University of East Anglia.

00:00:19 MD

All right, Caitlin, you know the form it's true or false. Pregnant women should not vape. True or false.

00:00:28 CN

If a pregnant woman is a smoker, she should absolutely vape rather than smoke, because we know that smoking is so dangerous. Then, by comparison, switching to using nicotine in a less harmful way, so through using e-cigarettes we know is far less harmful than continuing to smoke. And if vaping is a way that women manage to stay smoke-free then that’s a really, really positive health outcome for her and her family.

**Vaping is just swapping one addiction for another**

00:00:14 FN

Hi, I'm Felix Naughton. I'm a Professor of Health Psychology at the University of East Anglia, and I lead a research programme focused on looking at innovative ways to help people to quit smoking, particularly using new technologies.

00:00:29 MD

OK Felix. True or false? Vapers are just swapping one addiction for another.

00:00:33 FN

Well, that's partially true. But tobacco is a more difficult addiction to stop, and it's much more harmful than vaping. Vaping is much lower risk, unlike tobacco, which has additional constituents in it that make the product much more addictive.

00:00:51 MD

Felix Naughton, thank you very much.

**Nicotine vapes will not help you quit smoking**

00:00:09 MD

Does vaping help you quit smoking?

00:00:13 JHB

Hi, I'm Jamie Hartmann-Boyce, I'm an Assistant Professor in Health Policy and Management at the University of Massachusetts, Amherst, in the US. I also hold an honorary position in the Department of Primary Care Health Sciences at the University of Oxford in the UK.

00:00:29 MD

Jamie, true or false? Nicotine vapes won't help you quit smoking.

00:00:34 JHB

False. Evidence suggests that when you give people nicotine cigarettes and tell them they're there to help them quit smoking, it helps people quit smoking.

00:00:43 MD

Jamie, thank you very much. See you very soon.

**Vaping has no place on the NHS**

00:00:07 MD

Should we have vapes on the NHS?

00:00:13 IP

Hello. I'm Dr. Ian Pope. I research smoking and how to help people quit using e-cigarettes.

00:00:20 MD

True or false, vaping has no place on the NHS. True or false?

00:00:25 IP

False. Most people in England will interact with the NHS about 10 times over the course of the year, and those who interact are more likely to be people who smoke and therefore providing e-cigarettes on the NHS probably gives us the best opportunity to help people to quit.

00:00:43 MD

Thank you very much, Ian. See you later.

| **Supplementary Table 1. Interactions between vaping/smoking status and intervention in predicting vaping harm perceptions, adjusting for covariates.** | | |
| --- | --- | --- |
|  | Wald(3) | P value |
| Vaping is less harmful than smoking (vs. otherwise) | 2.94 | .401 |
| Vaping is as harmful as smoking: false | 3.61 | .307 |
| Vaping causes lung injury: false | 5.03 | .170 |
| Vaping causes cancer: false | 5.49 | .140 |
| When used in ways that does not involve smoking tobacco, nicotine is harmful: false | 5.22 | .156 |
| Vaping is just swapping one addiction to another: true | 1.05 | .789 |
| Nicotine vapes will not help you quit smoking: false | 3.85 | .279 |
| Vaping has no place on the NHS: false | 5.47 | .140 |

Interaction terms were added to the models described in Table 2 in the manuscript.
